# Common origin and somatic mutation patterns of composite lymphomas and leukemias

**DOI:** 10.1038/s41375-025-02549-y

**Published:** 2025-05-22

**Authors:** Victoria Berg, Anna Lollies, Markus Schneider, Patricia Johansson, Marc A. Weniger, Emma Albertini, Fabio Facchetti, Stefano Ascani, Abubakar Moawia, Susanne Bens, Anja Fischer, Reiner Siebert, Wolfram Klapper, Luisa Lorenzi, Enrico Tiacci, Sylvia Hartmann, Bettina Budeus, Martin-Leo Hansmann, Ralf Küppers

**Affiliations:** 1 Institute of Cell Biology (Cancer Research), Medical Faculty, University of Duisburg-Essen, Essen, Germany; 2 Department of Hematology and Stem Cell Transplantation, University Hospital Essen, University of Duisburg-Essen, Essen, Germany; 3 Pathology Unit, ASST Spedali Civili di Brescia, University of Brescia, Brescia, Italy; 4 Institute of Anatomic Pathology, University of Perugia and Hospital of Terni, Terni, Italy; 5 Institute of Human Genetics, Ulm University and Ulm University Medical Center, Ulm, Germany; 6 Department of Pathology, Hematopathology Section, University Hospital Schleswig-Holstein, Christian-Albrecht-University of Kiel, Kiel, Germany; 7 Institute of Hematology and Center for Hemato-Oncology Research (CREO), Department of Medicine and Surgery, University and Hospital of Perugia, Perugia, Italy; 8 Institute of Pathology, University Medicine Essen, Essen, Germany; 9 Frankfurt Institute of Advanced Studies, Frankfurt am Main, Germany; 10 German Cancer Consortium (DKTK), Essen, Germany; 11 Present Address: Gilead Science GmbH, Martinsried/Munich, Germany; 12 Present Address: Department of Pediatric Hematology and Oncology, University Children’s Hospital, University Hospital Essen, Essen, Germany

**Keywords:** Cancer genomics, Oncogenesis, Haematological cancer

## Abstract

When two lymphomas occur concurrently or sequentially in a patient, it is a major question whether they derive from the same lymphocyte or hematopoietic precursor cell or developed independently. We studied four composite classic Hodgkin lymphomas (HL) and other mature B-cell lymphomas, and two composite mature B- and T-cell neoplasias by whole exome sequencing (WES). Analysis of their IGV genes revealed that three composite B-cell lymphomas originated from common germinal center-experienced B cells. WES identified shared somatic mutations in the lymphomas of these clonally related composite lymphomas, indicating their derivation from a common, pre-malignant precursor. Most mutations were restricted to one or the other of these lymphomas, likely explaining how distinct lymphomas developed from a common ancestral B cell. In the two B-cell/T-cell lymphoma cases, and a composite clonally unrelated HL/chronic lymphocytic leukemia, the lymphoma partners did not share any somatic mutations. In three cases, we identified potentially oncogenic variants also in cells serving as constitutional controls. These variants may have contributed to development of a composite lymphoma/leukemia. We provide additional evidence of frequent clonal relation in composite lymphomas, highlight the multistep transformation process of related lymphomas with a likely pre-malignant intermediate common precursor, and support the importance of constitutional variants in lymphomagenesis.

## Introduction

In rare instances, two histopathologically distinct lymphomas occur concurrently in a patient, which is termed a composite lymphoma [1, 2]. Many composite lymphomas consist of a Hodgkin lymphoma (HL) and another mature B-cell lymphoma. Sequentially occurring histopathologically distinct lymphomas are also considered composite lymphomas in this study, because they have a similar pathogenesis as concurrent composite lymphomas [1]. It is a major question whether synchronous and metachronous composite lymphomas are a chance occurrence of two hematological malignancies in a patient, or whether they have a common origin. In more than half of the composite lymphomas consisting of a HL and another B-cell lymphoma investigated, the cases were clonally related and derived from the same mature B cells, as evidenced by common immunoglobulin (IG) V gene rearrangements [1, 3, 4]. If two distinct lymphomas derive from a common precursor, it is likely that the latter carried shared genetic lesions and hence represented a pre-malignant cell. Moreover, the lymphomas likely also carry distinct genetic lesions explaining the distinct histopathology of the lymphomas. This issue was so far addressed by a candidate gene approach for a few genes in a small number of cases [2, 5–7], revealing, for example, that the prototypical *BCL2*::*IGH* translocation was present in both partners in composite HL and follicular lymphoma (FL) [1, 2, 5, 8]. Recently, deep sequencing approaches indicated that composite lymphomas share some genetic lesions, but also carry distinct lesions [9–12]. However, these studies were restricted to selected gene panels, and with one exception performed on whole tissue sections or macrodissected areas, so that an unequivocal assignment of mutations to one and/or the other lymphoma remained uncertain.

In some types of lymphomas and leukemias representing transformed mature B or T cells [e.g., chronic lymphocytic leukemia (CLL), hairy cell leukemia], the first somatic mutation(s) can already occur in hematopoietic stem or precursor cells (HSC/HPC) [13–15]. Additionally, mutations in particular genes, such as *TET2*, can be found in descendants of clonally expanded HSC/HPC in elderly individuals [16]. Hence, combinations of a B- and a T-cell malignancy in a patient may also originate from a common HSC/HPC with somatic mutations. Furthermore, constitutional genetic variants might also contribute to the development of two lymphomas in one patient [17, 18].

In the present study, we performed whole exome sequencing (WES) of isolated lymphoma and non-tumor cells of four composite mature B-cell neoplasias, each with a classic HL as one of the partners, as well as two instances of composite mature B- and T-cell lymphoma to further clarify the complex somatic and constitutional genomic features underlying their pathogenesis, presenting these six cases as a starting point for further studies.

## Materials and methods

Materials and Methods are provided as supplementary material.

## Results

### Description of six cases of composite lymphomas

We studied four composite B-cell lymphomas and two combinations of B-cell and T-cell lymphoma (Table 1). Case 1 is a composite HL and CLL. Case 2 represents a sequential occurrence of a splenic marginal zone lymphoma (SMZL) and an HL appearing in a lymph node three years later. Case 3 is a composite HL and mantle cell lymphoma (MCL). In case 4, a FL was diagnosed, and three years later a HL was identified. In case 1, the Hodgkin and Reed-Sternberg (HRS) tumor cells (but not the CLL cells) were infected by Epstein-Barr virus (EBV) (not shown). In the three other HL, HRS cells were EBV-negative. Immunohistochemical stainings supported the diagnoses (Table 1 and Supplementary Figs. 1–3). In case 5, a CLL was initially diagnosed. Four months later, a T-prolymphocytic leukemia (T-PLL) appeared in the peripheral blood in addition to the still present CLL (Suppl. Fig. S4A). In case 6, a plasma cell leukemia (PCL) was first diagnosed, and two years later, an anaplastic large cell lymphoma (ALCL) and the PCL occurred concurrently in the peripheral blood (Supplementary Fig. 4B).

**Table 1:** Overview of composite lymphomas and leukemias.

Case	Lymphoma/leukemia	Age at diagnosis (years)	Gender	Concurrent or sequential	Tissue	Markers of tumor cells
1	HL and CLL	70	Male	Concurrent^a^	Lymph node (HL and CLL)	HRS cells: CD30^+^, CD20^−^, CD3^−^, PAX5^+^, EBV^+^
						CLL cells: CD20^+^, CD5^+^, CD23^+^, CD30^−^
2	HL and SMZL	67 (SMZL),	Male	Sequential	Lymph node (HL), spleen (SMZL)	HRS cells: CD30^+^, CD15^−^, PAX5^+^, CD20^−^, EBV^-^.
		70 (HL)				SMZL cells: CD20^+^, CD5^−^, CD23^−^, CCND1^−^
3	HL and MCL	85	Female	Concurrent	Lymph node (HL and MCL)	HRS cells: CD30^+^, CD19^−^, CD20^−^, PAX5^weak^, CD5^−^, CD45^−^, CCND1^−^, EBV^−^, *CCND1*::*IGH* translocation
						MCL cells: CD45^+^, CD19^+^, CD20^+^, CD5^-^, CCND1^+^, SOX11^−^, CD30^−^, *CCND1*::*IGH* translocation
4	HL and FL	61 (FL),	Female	Sequential	Lymph node (HL and FL)	HRS cells: CD30^+^, CD15^+/−^, CD20^−^, EBV^−^
		64 (HL)				FL cells: CD20^+^, BCL6^+^, CCND1^−^, BCL2^+/−^, CD10^+/−^
5	CLL and T-PLL	77	Female	Concurrent^b^	PBL	CLL cells: CD19^+^, CD5^+^, CD23^+^, Igκ^+^
						T-PLL cells: CD3^+^, CD4^+^, CD5^+^, CD8^−^
6	PCL and ALCL	73	Female	Concurrent^c^	PBL	PCL cells: CD38^+^, CD138^+^, cyIgλ^+^
						ALCL cells: CD3^+^, CD4^+^, CD30^+^, CD52^+^, ALK^−^

^a^First CLL diagnosis 2 years earlier.

^b^First, the CLL was diagnosed, and four months later both leukemias concurrently.

^c^PCL first diagnosed two years earlier.

### IGV gene analysis of composite B-cell lymphomas

In the four composite B-cell lymphomas, we first performed an analysis of the rearranged IGV genes of microdissected lymphoma cells to clarify whether the two lymphomas in the same patient were clonally related. The IGV genes were also analyzed for somatic mutations, as their presence indicates germinal center (GC) experience of the lymphoma precursor cell (Supplementary Fig. 5) [19].

In case 1, a HL and CLL, both lymphomas carried different IG heavy and light chain rearrangements, revealing that these lymphomas were derived from two clonally unrelated B cells (Table S4). The distinct lymphomas in cases 2, 3, and 4 each share a heavy chain rearrangement, and the lymphomas of cases 2 and 3 also have identical light chain rearrangements (Table S4). In case 4, no light chain was identified in the FL. This may, for example, be due to mutations in the primer binding site(s) which could impair binding and consequently amplification. All three lymphomas had mutated heavy and light chain rearrangements. The somatic mutations of the clonally related IGHV genes of cases 2, 3, and 4 and, for case 2, the IGKV genes, were identical, indicating that the somatic hypermutation process was largely silenced at the stage when the common lymphoma precursors started to develop into the distinct related lymphomas (Table S4).

Taken together, the IGV gene analysis of the four composite B-cell lymphomas revealed GC experience of the HRS cells in all instances, and a clonal relationship of the HRS cells to the other lymphoma in cases 2, 3, and 4. Conversely, the lymphomas of case 1 derive from two clonally unrelated B cells.

### WES of composite lymphomas

WES was performed on the six composite lymphomas/leukemias to identify shared and distinct somatic mutations. For cases 1, 2, 3, and 4, the lymphoma cells as well as non-tumor cells (NTCs) used as constitutional control were microdissected from frozen tissue sections, whereas the leukemic cells and NTCs in cases 5 and 6 were isolated by flow-cytometric cell sorting. We focussed on non-synonymous mutations, as these are more likely to be functionally relevant than silent mutations. The samples were sequenced at a mean depth of 63.3x (range: 30.6–154.6x). We considered as shared somatic mutations variants which were detectable in both tumor samples with a VAF of at least 10% and covered but absent (defined as VAF below 3%) in the NTC sample.

### Shared somatic mutations

The HRS and CLL cells of case 1, which did not share rearranged IGV genes, also did not share any somatic mutations. These lymphomas therefore were clonally unrelated and developed separately from each other.

**Fig. 1:**
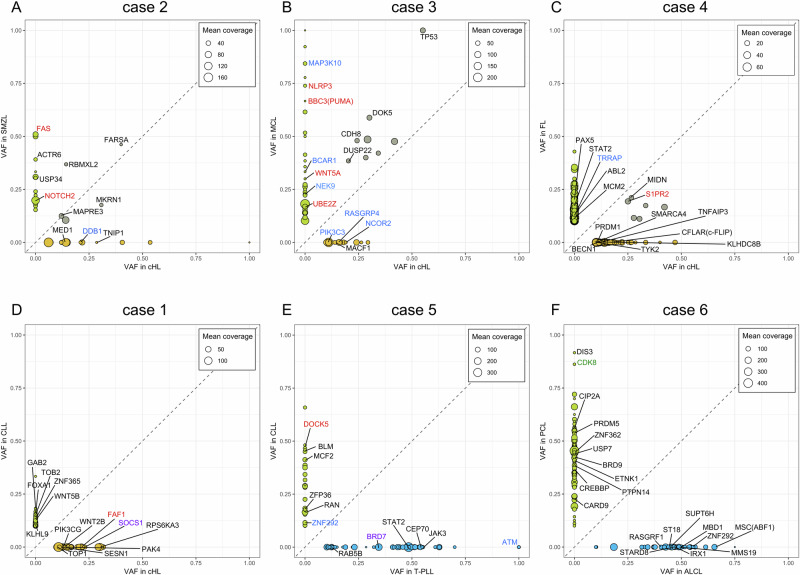
Shared and distinct non-synonymous somatic mutations in six cases of composite lymphomas. VAF of the mutation in the tumor partners is shown on the respective axes. Distinct mutations cluster on the X or Y axes, while shared mutations lie in between. The size of the dots corresponds with the mean coverage of the mutation site. Mutations in selected lymphoma-associated genes are labeled with the gene name. **A**–**C** display cases 2, 3, and 4, which each encompass clonally related lymphomas, as evidenced by both shared IGV gene rearrangements and shared somatic mutations, displayed in gray. **D**–**F** Display cases 1, 5, and 6, none of which share somatic mutations between their partner lymphomas. Distinct mutations in orange (HL), green (other mature B-cell lymphoma), or blue (T-cell lymphoma). Gene symbols marked according to mutation type: red: truncating mutation, blue: frameshift mutation, violet: in-frame insertion/deletion, black: missense mutation, green: splice site mutated. Mutations in IGV genes were excluded.

Shared mutations were found in the three composite B-cell lymphomas, which also shared IGV gene rearrangements, further proving that these lymphomas derive from a single mature B cell (Tables 2, 3, Fig. 1, and Supplementary Table S5). Case 2, the composite HL/SMZL, shared five mutations, all representing missense single nucleotide variants (SNVs). The gene phenylalanyl-tRNA synthetase alpha chain (*FARSA*) showed a clonal heterozygous mutation (VAF 40% in HL; 46% in FL). FARSA is involved in the pathobiology of MCL, where it is generally downregulated and also shows a tumor suppressor-like profile in diffuse large B-cell lymphoma [20]. MKRN1, also harboring a shared mutation in case 2, is a transcriptional co-regulator and E3 ubiquitin ligase [21]. It ubiquitinates proteins such as p53 and p21 and can be both pro- and anti-apoptotic [22]. It was not previously reported as recurrently mutated in hematological malignancies.

**Table 2:** WES analysis of composite lymphomas.

Case	Lymphoma	Non-synonymous mutations^a^	Distinct mutations	Shared mutations
1	HL	53	53	0
	CLL	66	66	
2	HL	15	10	5
	SMZL	19	14	
3	HL	25	17	8
	MCL	38	30	
4	HL	373	367	6
	FL	274	258	
5	CLL	31	31	0
	T-PLL	50	50	
6	PCL	102	102	0
	ALCL	55	55	

^a^Mutations in IG genes were excluded.

**Table 3:** Shared mutations.

Case	Gene	Position	Type of mutation	Protein effect	Tumor VAF	NTC VAF	Existing variant	SIFT	PolyPhen
2	RBMXL2	chr11:7089380	Missense	p.P87L	0.143/0.368 (HL/SMZL)	0.022	rs750554717, COSV60980351	Deleterious (0.04)	Possibly_damaging (0.634)
2	CA10	chr17:51633638	Missense	p.R268C	0.141/0.105 (HL/SMZL)	0.006	rs780641204, COSV53353383	Deleterious (0)	Probably_damaging (1)
2	FARSA	chr19:12924251	Missense	p.V470M	0.4/0.462 (HL/SMZL)	0	COSV53367759	Deleterious (0.04)	Probably_damaging (0.94)
2	MAPRE3	chr2:27025946	Missense	p.R231C	0.121/0.125 (HL/SMZL)	0		Deleterious (0)	Probably_damaging (0.998)
2	MKRN1	chr7:140456814	Missense	p.R275H	0.308/0.176 (HL/SMZL)	0	rs376798349	Tolerated (0.08)	Benign (0.066)
3	CDH8	chr16:61653967	Missense	p.D681H	0.243/0.48 (HL/MCL)	0.005		Deleterious (0.01)	Probably_damaging (1)
3	TP53	chr17:7673802	Missense	p.R273H	0.551/1 (HL/MCL)	0.018	rs28934576, CM920677, CM010472, CM004342, COSV52728930, COSV52676050, COSV52664805, COSV52660980	Tolerated (0.13)	Possibly_damaging (0.643)
3	CLTC	chr17:59666206	Missense	p.R587L	0.342/0.421 (HL/MCL)	0		Deleterious (0)	Possibly_damaging (0.613)
3	EML6	chr2:54871596	Missense	p.D779N	0.284/0.4 (HL/MCL)	0.007		Tolerated (0.08)	Possibly_damaging (0.786)
3	LMAN2L	chr2:96740032-96740034	In-frame deletion	p.A3del	0.418/0.476 (HL/MCL)	0.008			
3	DOK5	chr20:54591753	Missense	p.A183T	0.301/0.588 (HL/MCL)	0015	rs1485675129, COSV52821292	Deleterious (0.03)	Benign (0.001)
3	DUSP22	chr6:348938	Missense	p.T202M	0.203/0.385 (HL/MCL)	0.014	rs571363943, COSV100739786	Deleterious (0.01)	Benign (0)
3	GPR50	chrX:151181049	Missense	p.K489M	0.292/0.486 (HL/MCL)	0.025		Deleterious_low_confidence (0)	Possibly_damaging (0.536)
4	ALDH1A3	chr15:100900655	Missense	p.V322L	0.421/0.167 (HL/FL)	0.032	CM163655	Deleterious (0.01)	Probably_damaging (0.992)
4	MIDN	chr19:1255466	Missense	p.R301C	0.267/0.211 (HL/FL)	0	rs1362021177, COSV56295627	Deleterious (0)	Probably_damaging (0.994)
4	S1PR2	chr19:10224596	Nonsense	p.Q104*	0.25/0.194 (HL/FL)	0			
4	CYP27C1	chr2:127206078	Missense	p.R99W	0.333/0.174 (HL/FL)	0		Deleterious (0)	Possibly_damaging (0.864)
4	CNTLN	chr9:17135288-17135289	Frameshift insertion	p.A75Dfs*48	0.278/0.115 (HL/FL)	0			
4	CFP	chrX:47626142	Missense	p.T387N	0.304/0.111 (HL/FL)	0		Tolerated (0.33)	Benign (0.335)

In case 3, the composite MCL and HL share eight somatic mutations. These include a probably originally heterozygous mutation in the tumor suppressor gene *TP53*, where the other allele may have been deleted in the MCL, resulting in a hemizygous mutation (55% VAF in HL, 100% VAF in MCL). This *TP53* hotspot mutation (rs28934576, p.R273L) [23] is associated with pathogenicity in various cancers, including B-cell lymphomas [24]. *TP53* mutations have been identified in HRS cells before [25, 26], and are frequent in MCL [27]. *DUSP22*, a known tumor suppressor gene in lymphomas, is mutated in both lymphomas as well.

The composite HL and FL in case 4 shared six mutations, one of them being a nonsense mutation in *S1PR2* (25% VAF in FL, 19% VAF in HL), a tumor suppressor gene commonly silenced in aggressive B-cell lymphomas [28–30]. A further mutated gene, midnolin (*MIDN*), facilitates the ubiquitin-independent proteasomal degradation of a number of transcription factors that are known as oncogenic, such as FOSB [31] and EGR1, indicating a possible role in lymphomagenesis.

In cases 5 and 6, the two composite B- and T-cell lymphomas, no shared mutations were identified, showing that these lymphomas were not clonally related.

### Distinct somatic mutations in composite lymphomas

In addition to the shared somatic mutations, all lymphomas in this study also carried distinct mutations, i.e., mutations present in only one of the tumors and absent also in the NTC samples (Tables 2, 4, and Supplementary Table 6). These mutations occurred after the lineages of the lymphomas diverged. Each lymphoma harbored between 9 and 367 non-synonymous distinct mutations (Table 2). Case 4, with 367 (HL) and 258 (FL) distinct somatic mutations, likely represents hypermutating lymphomas. In all cases, mutations in lymphoma-associated genes were identified.

**Table 4:** Selected unique mutations.

Case	Lymphoma	Affected gene	Position	Type of mutation	VAF (%)	Protein effect	SIFT/PolyPhen	Existing variants
1	HL	*SOCS1*	chr16:11255132	Missense	14.3	p.S116N	Deleterious/probably damaging	COSV59659400
1	HL	*SOCS1*	chr16:11255286-11255297	In-frame deletion	14.9	p.H61_Y64del		
1	HL	*SOCS1*	chr16:11255303	Missense	14.9	p.R59P	Tolerated/benign	
1	HL	*FAF1*	chr1:50788078	Premature Termination	31.6	p.Q97*		
2	SMZL	*NOTCH2*	chr1:119915524	Activating truncation	20	p.R2400*		
2	SMZL	*FAS*	chr10:89014284	Premature termination	50	p.W281*		COSV58238798
2	HL	*TNIP1*/*ABIN1*	chr5:151035688	Missense	58.3	p.D472V	Deleterious/probably damaging	
3	MCL	*BCAR1*	chr16:75235905-75235906	Frameshift insertion	33.3	p.A378Rfs*9		
4	HL	*TNFAIP3*	chr6:137880193	Missense	10.5	p.C677R	Deleterious/probably damaging	
4	HL	*CFLAR*/*c-FLIP*	chr2:201129872	Missense	13	p.A3S	Deleterious/possibly damaging	
4	HL	*CFLAR*/*c-FLIP*	chr2:201129878	Missense	13	p.V5F	Deleterious/probably damaging	
4	HL	*KLHDC8B*	chr3:49175078	Missense	11.1	p.L261F	Tolerated/possibly damaging	
4	FL	*STAT2*	chr12:56343472-56343473	Missense	16.7	p.G825C		rs1555169006
4	FL	*PAX5*	chr9:36840630	Missense	10	p.P369L	Deleterious/probably damaging	rs776430331
4	HL	*PRDM1*	chr6:106106420	Missense	10.5	p.C622F	Deleterious/probably damaging	
5	T-PLL	*STAT2*	chr12:56349034	Missense	48.7	p.P489H	Deleterious/probably damaging	rs138681270
5	T-PLL	*JAK3*	chr19:17838299	Missense	55.1	p.M511I	Tolerated/benign	rs752661478, COSV71685384, COSV71685422, COSV71685783
5	T-PLL	*ATM*	chr11:108299769-108299791	Frameshift deletion	100	p.I1688Lfs*6		
5	CLL	*BLM*	chr15:90765337	Missense	45	p.S706P	Deleterious/benign	
5	CLL	*DOCK5*	chr8:25408099	Premature termination	48.1	p.W1737*		
6	PCL	*DIS3*	chr13:72761792	Missense	91.7	p.R789W	Deleterious/possibly damaging	rs961788436, COSV66707898
6	PCL	*CREBBP*	chr16:3757885	Missense	36.8	p.C1178Y	Deleterious/probably damaging	
6	ALCL	*MSC*	chr8:71843833	Missense	66	p.E116K	Deleterious/probably damaging	

In case 4, the HL prominently carried somatic mutations in HL-related genes, such as *TNFAIP3*, *CFLAR*/*c-FLIP,* and *KLHDC8B* [32–35]. *SOCS1*, one of the most frequently mutated genes in HL [36], was mutated in three different positions in the HRS cells of case 1. Other affected genes in the HRS cells with implications in lymphomagenesis include *FAF1* [37] (case 1), *TNIP1*/*ABIN-1* [38] (case 2), and *PRDM1*/*BLIMP1* [39, 40] (case 4). The non-Hodgkin lymphoma partners of cases 1–4 all also carried mutations in lymphoma-related genes (Table 4).

For the B-cell/T-cell lymphoma combinations, typical mutations were also identified, such as a mutation in *DIS3* in the PCL [41], or a mutation of *MSC* (p.E116K) in ALCL of case 6 [42].

Taken together, we identified numerous mutated genes with roles in lymphomagenesis which likely contributed to the development of the lymphomas.

### Somatic copy number variations and a chromosomal translocation

**Fig. 2:**
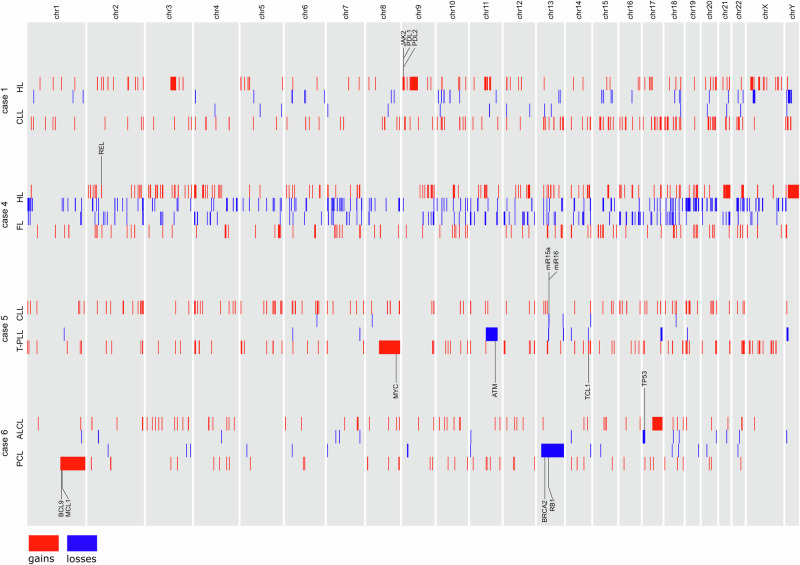
Copy number variations in four cases of composite lymphomas. CNVs in cases 1, 4, 5, and 6 are displayed. Gains are marked in red, losses are marked in blue. Oncogenes and tumor suppressor genes of interest that are affected by CNVs are marked. CNVs smaller than 50 kb were excluded in the figure.

We also performed an analysis of copy number variations (CNVs) (Fig. 2). The analysis of CNVs in WES data, as performed here, is mostly restricted to large CNVs. Accordingly, we focused our analysis on large events and selected hotspots for CNVs. In cases 2 and 3, no reliable CNV data were obtained, likely because of issues with data quality due to whole gene amplification used in these cases.

We identified several CNVs that are known recurrent events in HL, including an amplification of *JAK2* and the neighboring *PD-L1* and *PD-L2* genes (9p23-p24) in the HL of case 1, and a *REL* amplification (2p13) in the HL of case 4 (Fig. 2, and Supplementary Fig. 6).

The T-PLL possesses a large deletion in 11q (including *ATM*), a gain of 8q (including the oncogene *MYC*), and an amplification of *TCL1A* and *TCL1B* (14q32), which are all hallmarks of T-PLL. The CLL partner lost a region of 13q14, an event shared with more than 50% of CLL cases [43]. In case 6, the ALCL lost a large part of 17p, including *TP53*. The PCL had gains in 1q, as is typical for plasma cell malignancies and lost 13q, which encompassed the tumor suppressor genes *BRCA2* and *RB1*.

Since WES data cover only parts of the genome, they are not suitable for in silico breakpoint analyzes. We therefore performed FISH analyzes to identify translocations. Indeed, the clonally related HL/MCL carried an identical *IGH*::*CCND1* translocation as confirmed by CD30-FICTION (not shown). Conversely, both partners of the HL/FL composite lymphoma were negative for the *IGH*::*BCL2* translocation typical of classic FL. The FL did not carry a mutation in *STAT6*, which has been reported to be present in 55–80% of *IGH::BCL2*-negative FL [44, 45]. However, we detected a 16p13 deletion in this FL (Fig. 2), and this event is enriched in *IGH::BCL2*-negative FL (seen in 39% of *IGH::BCL2*-negative, but only 9% of translocation-positive FL) [44].

### Candidate constitutional variants or rare germline polymorphisms

The analysis primarily focused on somatic mutations present in one or both of the lymphomas, but missing in the NTCs. However, constitutional polymorphisms or variants may also contribute to the pathogenesis of composite lymphomas [46, 47]. To study the potential contribution of constitutional variants or rare polymorphisms in the development of composite lymphomas, we performed an analysis of genetic variants present in the lymphoma cells as well as the NTCs.

We define constitutional events as non-synonymous genetic variants that are shared between both tumors and the NTCs at high VAFs (≥30% in both tumors and NTCs). These include constitutional polymorphisms that were not excluded by the panel of normal filtering, such as novel mutations, extremely rare alleles, or mutations in hematological cells.

As expected, non-tumor-shared events were identified in all cases analyzed (Supplemantary Table S7), with 400–653 events per case, case 2 being an outlier with only 67 events. While most of these represent unrelated constitutional polymorphisms or somatic mutations in non-cancer genes, a small number of events in each case were found in lymphoma-related genes. Some of these events are specifically related to lymphomas, such as in case 5, which includes nine single-nucleotide events associated with lymphomas, e.g., the p.P925T missense variant in *EP300* (COSV54337074) [48, 49], or events in *MCL1* and *PTPN13* (Table 5).

**Table 5:** Selected constitutional variants.

Case	Affected gene	Position (hg38)	Type of mutation	Protein effect	VAF in NTCs	Existing variants	ACMG-AMP criteria and classification	CADD	GnomAD allele frequency	AlphaMissense
1	*CD27*	chr12:6445520	Missense	p.R78G	0.469	rs145433356	VUS	23.8	0.0001	0.598 (likely pathogenic)
1	*CREBBP*	chr16:3781229	Missense	p.L551I	0.644	rs61753381, COSV52134580	Benign	24.3	0.007154	0.087 (likely benign)
1	*CREBBP*	chr16:3729277	Missense	p.V1924M	0.687	rs368145743	Likely benign	23.9	0.000445	0.095 (likely benign)^c^
1	*FANCA*	chr16:89771678	Missense	p.M717I	0.491	rs1131660	Benign	23.7	0.022344	0.176 (likely benign)
1	*GATA2*	chr3:128486117	Missense	p.P161A	0.538	rs34799090, COSV62007567	Benign	19.35	0.008537	0.07 (likely benign)
1	*MYC*	chr8:127740678	Missense	p.S362F	0.464	rs200431478, COSV99420567	Likely benign	27.8	0.000335	0.414 (ambiguous)
1	*SETD2*	chr3:47120671	Missense	p.R1322Q	0.468	rs147170912, COSV99042141	VUS	22.7	0.000007	0.103 (likely benign)
1	*TET2*	chr4:105275662	Missense	p.V1718L	0.545	rs142312318, COSV54398060	Likely benign	0.254	0.004477	0.098 (likely benign)
1	*TRAF3IP2*	chr6:111566485	Missense	p.E479K	0.391	rs149504543	VUS	21.3	0.000093	0.141 (likely benign)
1	*JAK3*	chr19:17834887	Missense	p.V722I	0.475	rs3213409, CM000172, COSV71685519	Benign	14.82	0.007432	0.071 (likely benign)
2	*DUSP22*	chr6:304648	Missense	p.I14M	0.419	COSV60528266	VUS	21	no information	0.564 (likely pathogenic)
2	*NOD2*	chr16:50729867-50729868	Frameshift insertion		0.333		VUS	34	no information	no information
3	*ARID1A*	chr1:26763012	Missense	p.N820S	0.555	rs773242876, COSV99055395	VUS	23.8	0.000007	0.086 (likely benign)
4	*BRCA2*	chr13:32398184-32398185	Frameshift insertion	p.Y3225IfsTer30	0.333	CI022172	Pathogenic	28.5	no information	no information
4	*ATM*	chr11:108335880	Missense	p.Q2729H	0.382	rs587781946, CM143104, COSV53762213, COSV99586681	VUS	23.7	0.000016	0.976 (likely pathogenic)
4	*BTK*	chrX:101390479	Missense	p.E24G	0.706	rs5951308, COSV58121598	Benign	4.826	0.338641	0.996 (likely pathogenic)
4	*NOTCH2*	chr1:119925578	Missense	p.L1413H	0.4	rs41313282, COSV56681418	Benign	19.01	0.003746	0.07 (likely benign)
4	*SETD2*	chr3:47122481	Missense	p.N719D	0.357	rs115859828, COSV57453200	VUS	23	0.00005	0.134 (likely benign)
5	*ATM*	chr11:108254034	Missense	p.S707P	1	rs4986761, CM013692, COSV53743430	Benign	11.04	0.007818	0.08 (likely benign)
5	*BRCA1*	chr17:43092412	Missense	p.S1040N	0.371	rs4986852, CM940175, COSV58791858	Benign	14.02	0.013154^a^	0.119 (likely benign)
5	*BRCA1*	chr17:43094045	Missense	p.R496C	0.530	rs28897676, CM065005, COSV58797441	Benign	4.7	0.000235^a^	0.08 (likely benign)
5	*IBTK*	chr6:82191202	Missense	p.H1134R	0.426	rs145495116	Likely benign	15.48	0.000513	0.07 (likely benign)
5	*MCL1*	chr1:150578851	Missense	p.A227V	0.555	rs11580946, COSV57191585	Benign	25.9	0.008742	0.919 (likely pathogenic)
5	*NFKB1*	chr4:102597543	Missense	p.M507V	0.5	rs4648072	Benign	8.694	0.018841	0.082 (likely benign)
5	*PTPN13*	chr4:86809827	Missense	p.T2386I	0.392	rs61730641, COSV57419424	Benign	24.1	0.012955^b^	0.665 (likely pathogenic)
5	*PTPN13*	chr4:86803817	Missense	p.R2210Q	0.449	rs61750816, COSV57401812	Benign	18.28	0.021763	0.071 (likely benign)
5	*EP300*	chr22:41150154	Missense	p.P925S	0.667	rs148884710, COSV54339374, COSV54337074	VUS	20.3	0.004099	0.099 (likely benign)
6	*KMT2C*/*MLL3*	chr7:152163145	Missense	p.Q3478E	0.413	rs142835638, COSV51503272	Benign	22.8	0.00303	0.121 (likely benign)
6	*MTOR*	chr1:11247950	missense	p.A329T	0.568	rs35903812, COSV63870597	Benign	23.4	0.002625	0.086 (likely benign)
6	*NOD2*	chr16:50722629	Missense	p.G908R	0.627	rs2066845, CM011829	Benign	26.3	0.01424^b^	0.85 (likely pathogenic)^d^
6	*S1PR2*	chr19:10224876	Missense	p.N10L	0.5	rs56357614	Benign	16.37	0.008214	0.385 (ambiguous)

^a^GnomAD_Exome.

^b^ALFA.

^c^Recurrently mutated in AML https://pmc.ncbi.nlm.nih.gov/articles/PMC6471684/.

^d^Risk of Crohn’s disease.

Case 1 harbors an activating missense mutation in *JAK3* (p.V722I, COSV71685519) that is related to various types of lymphomas and leukemias, and found recurrently in other cancers as well [50, 51]. This constitutively phosphorylated mutant protein induces cytokine-independent cell growth in the murine cell line Ba/F3, indicating a transforming effect [52, 53]. In all six cases, SNVs were detected in well-known tumor suppressors and oncogenes or B cell- and lymphoma-associated genes (Table 5). Some of these variants were detected as somatically mutated in cancers according to the COSMIC database, often in a neoplasm of the hematopoietic lineage.

Additionally, variants were processed with an artificial intelligence-based variant prioritization platform to interpret whether these events represent “true” constitutional events linked to lymphomagenesis, cancer predisposition, and/or immune dysfunction. This analysis identified a pathogenic *BRCA2* mutation (c.9672dup, p.Tyr3225IlefsTer30) in case 4 as a likely contributor to a cancer predisposition syndrome, possibly intensified by a variant of unknown significance in *ATM* (c.8187A > T, p.Gln2729His) (Table 5). These DNA damage repair deficiencies might explain why both lymphomas in case 4 show unusually high mutational frequencies.

## Discussion

### Composite lymphomas consisting of a classic HL and another B-cell lymphoma are frequently clonally related and derive from GC-experienced B cells

In the present study, we analyzed six cases of composite lymphomas regarding their shared or distinct origin. We confirmed previous results that concurrent or sequential combinations of an HL and another B-cell lymphomas are frequently clonally related [1–3], as evidenced by shared IGV gene rearrangements in three of the four composite lymphomas analyzed here. These shared rearrangements were all somatically mutated, indicating that the separation into two different lymphomas happened after the progenitor B cells gained GC experience. Case 1, a composite HL and CLL, was the only B-cell composite lymphoma in our study that was clonally unrelated. The CLL carried unmutated IGV genes suggesting a pre-GC B-cell derivation, whereas the HRS cells carried somatically mutated IGV genes, in line with the known GC derivation of HL [54].

### Shared somatic mutations drive tumorigenesis

In all three cases with shared IGV gene rearrangements (cases 2, 3, and 4), we also identified shared somatic mutations in the WES analysis, further corroborating that the lymphoma partners derive from one cell of origin. Many of these mutations were found in lymphoma-related genes such as *TP53*, *DUSP22*, and *S1PR2*. These may represent driver mutations, such as a known destructive mutation in *TP53* in case 3, or less deleterious events that only “nudge” the cells towards becoming cancerous.

The numbers of shared non-synonymous somatic mutations in the clonally related composite lymphomas were relatively low, with only 5, 8, and 6 of these mutations in cases 2, 3, and 4, respectively (Table 2). The number of distinct mutations was higher in each case. This indicates that the common precursor of both lymphomas was a pre-malignant cell with relatively few mutations and that most mutations happened after the two separate lymphoma precursors had developed. Multiple distinct mutations may be needed to generate an HL versus another B-cell lymphoma from a GC B cell that was the origin of both lymphomas. However, we likely underestimate the number of shared mutations, as WES from pools of microdissected cells does not cover the exome completely at sufficient sequencing depth, and mutations outside the exome were not covered.

### Distinct somatic mutations determine the lymphoma type in composite lymphomas

Distinct mutations that are only present in one lymphoma partner contribute to the differentiation between both lymphomas, as evidenced by finding entity-typical mutations in the lymphomas. In addition to these mutations, we also analyzed large CNVs in cases 1, 4, 5, and 6. The majority of distinct CNVs we observed were hallmarks of the respective lymphoma subtype, e.g., 8q and 11q deletions in the T-PLL, a 13q14 deletion in the case 5 CLL, and the gains of *JAK2*/*PD-L1*/*PD-L2* and *REL* in the HL of cases 1 and 4, respectively. All of these events likely contributed to the differentiation into a specific lymphoma type.

### Lymphoma-related constitutional SNVs might contribute to multi-step lymphomagenesis

In all cases analyzed, we identified sequence variants that were shared between both lymphomas and the NTCs of a given case, which may represent constitutional variants. We classified constitutional variants according to the ACMG criteria and with few exceptions, most of them were called (likely) benign. Among the exceptions was a likely pathogenic mutation in the DNA damage repair gene *BRCA2* in case 4. We also screened the literature for variants that were called (likely) benign, but had some measure of functional data that indicated a role in lymphomagenesis, and identified, e.g., a variant in *JAK3* that is scored as benign by all algorithms we employed, but is nonetheless functionally relevant in lymphomagenesis due to it being a phosphomimetic mutation that constitutionally activates JAK3. This discrepancy underscores the fundamental differences of in silico approaches to variant classification in the constitutional (germline) versus the tumor (somatic) setting and a need to functionally validate candidate variants in the respective context. It is also intriguing that many of the (likely) benign variants affect commonly altered pathways or complexes in lymphoma. Thus, one could speculate that they might contribute to lymphomagenesis via a kind of polygenic or low-penetrance predisposition.

We also identified variants in multiple genes that are often mutated in clonal hematopoiesis [55]. However, since these events all had VAFs higher than 35% in both lymphomas and NTCs, it is unlikely that these events represent clonal hematopoiesis, as in that case, one would expect much lower VAFs in the NTCs.

Some possibly oncogenic constitutional events that are shared between lymphomas and NTCs might contribute to cancer development later on, analogous to tumor predisposition syndromes. Genetic predisposition to lymphomas is well-known [56] and has been described in the context of familial increased risk of lymphoma/leukemia as well [47]. As far as we know, none of the patients included in this study was clinically diagnosed with a tumor predisposition syndrome, but the development of multiple distinct lymphomas that are not clonally related might hint at a common constitutional predisposition.

Our data add to the growing evidence that constitutional or early somatic events can contribute to tumorigenesis [46] and have implications for the genesis of both singular and composite lymphomas: the first hit(s) of a composite lymphoma can happen early during hematopoiesis or might even be a constitutional variant, as is the case for singular lymphomas as well.

### Two models of multi-step lymphomagenesis in composite lymphomas

**Fig. 3:**
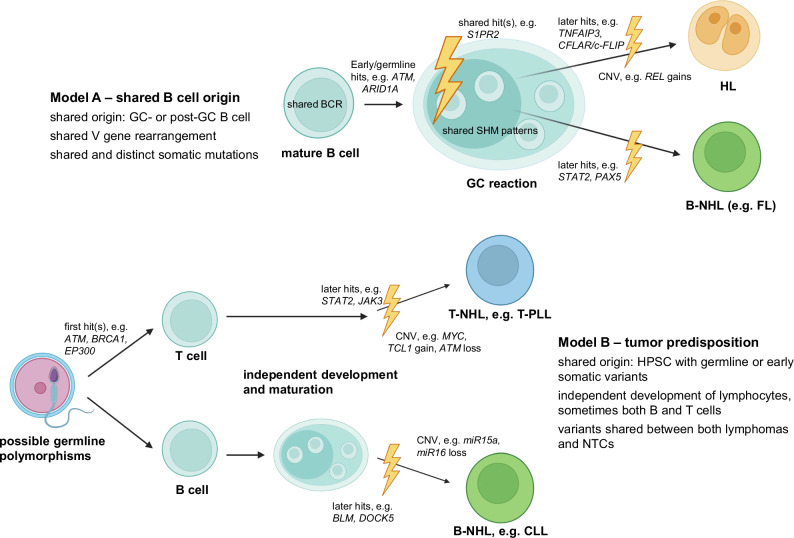
Models of lymphomagenesis in composite lymphomas. Model A depicts tumorigenesis in lymphomas with a GC-experienced B cell as a shared origin, with case 4, a composite HL/FL, as an example. B shows a model of tumor predisposition that includes shared constitutional/HSC variants, with case 5, a composite CLL/T-PLL, as an example. Other composite lymphomas may have no relation and be a chance occurrence. Figure created with biorender.com.

Based on our data, we propose two models of multi-step lymphomagenesis in composite lymphomas (Fig. 3): Model A, representing GC-derived lymphomas, is based on cases 2, 3, and 4, all of which were composite B-cell lymphomas sharing mutated IGV gene rearrangements and somatic mutations. These cases likely represent a “typical” B-cell lymphomagenesis originating in the GC reaction due to factors such as rapid cell division and DNA damage during somatic hypermutation. These processes can generate pre-malignant cells, which may later split into two different lymphoma entities. A common GC B-cell derivation is indeed seen in nearly all cases of clonally related composite B-cell lymphomas [1].

The as-of-yet speculative model B is based on cases 1, 5, and 6. These cases do not share an IGV gene rearrangement or somatic mutations but have some early candidate transforming events in common with NTCs. We regard these cases as possible examples of a “predisposition” model, in which oncogenic lesions would occur early during hematopoietic differentiation or are present as constitutional variants, predisposing the patient to develop multiple lymphomas.

These different mechanisms of pathogenesis may be combined, such as in case 4, which had both shared somatic mutations and a constitutional variant of *BRCA2*, which predisposes to cancer. In addition to the two models, there are likely also cases with completely unrelated composite lymphomas, where the patient is not predisposed to lymphomagenesis either.

As our analysis was restricted to exonic SNVs and large CNVs, analyzing additional dimensions, such as environmental factors, immunodeficiencies, epigenetic changes, mutations in areas not covered by WES, or therapy effects in sequential lymphomas, may also shed more light on the development of composite lymphomas.

### Study limitations

Our approach is limited primarily by the low sample availability since composite lymphomas are rare and we needed to obtain samples from both lymphoma partners as frozen material to perform laser microdissection. Furthermore, all six cases consist of different lymphoma entities, further limiting cross-case comparisons. There is also a lack of “true” constitutional sequencing information from a non-hematopoietic tissue. The comparatively shallow mean sequencing depth of 63.3 limits the analysis of subclonal mutations, so we focused on clonal mutations. Working with WES data also limits our approach to exonic variants, which means that, e.g., shared mutations in regulatory regions would not have been identified. We did not only identify variants that are already linked to lymphomagenesis, but a large number of candidate variants as well. Functional studies will be needed to clarify the relevance of these variants.

## Supplementary information

Supplementary Methods and Data

Supplementary Table S6

## Data Availability

Curated constitutional variants of each patient and somatic mutations in the lymphomas are available in Supplementary Tables S5– S7. The raw exome sequencing data of this study will be made available through the European Genome-Phenome Archive (EGA).
