# Supplementary material for: Common origin and somatic mutation patterns of composite lymphomas and leukemias
Source: Leukemia. 2025 May 22;39(8):1960–71. doi: 10.1038/s41375-025-02549-y (PMC12310529; doi:10.1038/s41375-025-02549-y)
Supplement: Supplementary file 1 — Supplementary Methods and Data [file 41375_2025_2549_MOESM1_ESM.docx]

**Supplement for the article:**

**Common origin and somatic mutation patterns of composite lymphomas and leukemias**

by Victoria Berg, Anna Lollies, et al.

**Content**

Supplementary Methods

Supplementary Figure S1. Histological evaluation of case 1, a composite CLL and classic HL

Supplementary Figure S2. Histological evaluation of case 2, a composite SMZL and classic HL

Supplementary Figure S3. Histological evaluation of case 3, a composite MCL and classic HL

Supplementary Figure S4. Flow-cytometric sorting strategies of combined B-NHL/T-NHL cases

Supplementary Figure S5. IGV gene sequences of combined lymphomas

Supplementary Figure S6. Copy number variations in composite lymphoma cases

Suppl. Table S1: Antibodies used for Laser microdissection immunohistochemistry

Suppl. Table S2: Antibodies used for flow cytometry

Suppl. Table S3: Antibodies used for diagnostic immunohistochemistry

Suppl. Table S4: IGV gene analysis of composite B-cell lymphomas.

Suppl. Table S5: Full list of all shared mutations.

Suppl. Table S7: Full list of all constitutional variants.

supplied as separate file:

Suppl. Table S6: Full list of all distinct mutations.

**Supplementary Materials and Methods**

**Patients and biopsy material**

Tumor material from four patients with composite HL and B-NHL and two instances of combined B-NHL and T-NHL were available for this study. The study was approved by the local ethics committees of the University Hospitals of Frankfurt/Main (8/15) and Essen (15-6185-BO). Frozen lymph node or spleen diagnostic biopsy specimens were available for the composite lymphomas, and peripheral blood samples were used for the two combined leukemic B-NHL and T-NHL.

**Immunohistochemistry staining**

The antibodies that were used for IHC stainings of the lymphomas are listed in Suppl. Table S3. Staining was performed by standard methods.

**RNAscope *in situ* hybridization**

To localize kappa and lambda light chain transcripts by RNAscope assay (Advanced Cell Diagnostics, Newark, CA, USA) we used RNAscope 2.5 HD Assay-RED, RNAscope® Probe-Hs-IGL (cat n. 312561) and RNAscope® Probe-Hs-IGK (cat n. 312551). Briefly, freshly cut 2 μm sections were deparaffinized in xylene and treated with the peroxidase block solution for 10 min at room temperature followed by the retrieval solution for 15 min at 98°C and by protease plus at 40°C for 30 minutes. The hybridization was performed for 2 hours at 40°C. The signal was revealed using RNAscope 2.5 HD Detection Reagent and Fast Red [1].

**FISH and FICTION analyses**

Interphase cytogenetic studies for the detection of *IGH*::*CCND1* and *IGH*::*BCL2* translocations on formalin-fixed, paraffin-embedded sections of the cases of combined cHL/MCL (case 3) and cHL/FL (case 4), respectively, were performed as described recently [2]. We applied commercially available double-color double fusion (LSI IGH/BCL2 DCDF, LSI IGH/CCND1 DCDF) and break-apart probes (LSI IGH BAP, LSI BCL2 BAP, LSI CCND1 BAP, all from Abbott, Chicago, IL, USA). For detection of the respective changes in HRS cells, immunofluorescence with an anti-human CD30 (Ber-H2) monoclonal antibody (Dako, Glostrup, Denmark) and a F(ab')2-Rabbit anti-mouse IgG (H+L) Cross-Adsorbed Secondary Antibody, Alexa Fluor 594 (Thermo Fisher Scientific (Invitrogen/Life Technologies), Waltham, MA, USA) was performed according to the FICTION technique [3]. 50 CD30-positive and 100 CD30-negative cells for the cHL/MCL sample and 100 cells from the FL section of the cHL/FL case were evaluated under a fluorescence microscope (Zeiss, Oberkochem, Germany) by at least two independent observers and documented using the ISIS imaging software V5.8.7 (MetaSystems, Altlussheim, Germany).

**Microdissection of HRS and NHL cells**

Lymphoma and non-lymphoma cells were microdissected from 7 µm thick frozen tissue sections of lymphoma biopsies and mounted on 1.0 mm PEN membrane-covered slides (PALM, Zeiss, Jena, Germany). Anti-CD30-staining (BerH2, Dako/Agilent, Santa Clara, CA, USA) or hemalaun and eosin staining was performed for detection of HRS cells. B-NHL cells were selected based on additional immunohistochemical stainings and counterstaining with hematoxylin (Suppl. Table S1). Primary antibody incubation was performed for 1 h at room temperature. The Super Sensitive™ Link-Label IHC Detection System (BioGenex, Fremont, CA, USA) was used for visualization, employing streptavidin-conjugated alkaline phosphatase chemistry and using DAKO liquid permanent red (Agilent) as a color substrate. Approximately 3000 lymphoma cells and non-tumor cells (NTCs) each were isolated for WES using the Zeiss PALM pressure catapulting laser microdissection system (Zeiss, Oberkochen, Germany). Single CD30^+^ HRS cells and groups of 3 to 5 NHL cells were laser-microdissected.

IGV gene analysis was performed on single or up to 10 tumor cells and NTCs, which were isolated using the PALM laser capture microdissection technique as described previously [4], and collected in 20 µl Q5 PCR reaction buffer (New England Biolabs, Ipswich, MA, USA), supplemented with 0.1% Triton X-100. Small pieces of membrane distant from tissue sections were catapulted into buffer and used as negative controls in the PCR in addition to buffer-only negative controls.

**IGV gene analysis of isolated lymphoma cells**

Rearranged IGHV and IGKV genes were amplified from the microdissected lymphoma cells by seminested PCR with framework region I group-specific primers and IGHJ and IGKJ primers as described [4]. PCR products were isolated from an agarose gel after electrophoresis and sequenced by Sanger sequencing on an ABI 3130 Genetic Analyzer (Applied Biosystems, Waltham, MA, USA). Sequences were evaluated with the IMGT/V-Quest software (http://www.imgt.org/IMGT_vquest/input, version 3.6.3). Additionally, WES data were analyzed with the Python program IgCaller v1.4-beta, which can identify IGV gene rearrangements in next generation sequencing (NGS) data of lymphomas to verify and expand upon the data generated *via* IGV gene PCR.

**Isolation of lymphoma and leukemia cells by flow cytometry**

In the combined ALCL/PCL and CLL/T-PLL, peripheral blood mononuclear cells (PBMCs) were isolated from whole blood with a density gradient centrifugation. ALCL cells were then identified and sort-purified as CD30^+^CD4^+^CD3^+^ cells. In a separate staining, PCL cells were isolated as CD138^+^CD14^-^ cells. Monocytes, used as NTC controls and defined as CD138^-^CD14^+^ cells, were also sort-purified. In the combination of T-PLL and CLL, T-PLL cells were sorted as CD5^+^CD19^-^ cells, and CLL cells as CD5^+^CD19^+^ cells. In this case, CD16^+^ granulocytes were isolated as non-tumor control. Sort-purification was performed with a FACS-ARIAIII cell sorter (BD Biosciences, Heidelberg, Germany). Antibodies used for cell sorting are listed in Suppl. Table S2. The sorting strategies are presented in Suppl. Figure S4.

**Isothermal whole genome amplification and WES**

DNA of the two cases of combined B-cell/T-cell malignancies was isolated using the QIAamp DNA Micro Kit (Qiagen, Hilden, Germany). DNA of laser-microdissected cells was extracted by isopropanol precipitation after proteinase K digestion. Whole genome amplification (WGA) was performed on cases 2 and 3 using the Qiagen REPLI-g kit (Qiagen) to compensate for low cell numbers (this was omitted for cases 1 and 4). For this, DNA from isolated lymphoma cells (and from the NTCs) was aliquoted into three tubes, and separate libraries were generated and sequenced for each aliquot. Exome sequences were captured using NimbleGen SeqCap EZ Choice kit (Roche, Basel, Switzerland) and libraries were prepared using NEBNext Ultra DNA Library Prep Kit (New England Biolabs, Ipswich, MA, USA) Sequencing was performed at the DKTK core facility in Heidelberg.

**Evaluation of WES data**

Raw reads were trimmed with TrimGalore v0.6.6 and aligned to hg38 with bwa and standard settings for each tumor and normal sample separately. Duplicates were marked with MarkDuplicates from Picard Tools v2.8.1. Variant calling was performed using mutect2 from GATK v4.2.6.1 with LearnReadOrientationModel to be able to identify possible errors during sample preparation, processing each lymphoma separately. In case a WGA was performed, only mutations found in at least 2 of 3 independently sequenced aliquots, but not in the NTCs, were included. The resultant VCF files were compared to identify shared mutations. Only variants that passed the filtering criteria of mutect2 were considered. Variants with a sequencing depth of less than 10 in the tumor cells or NTCs were excluded. Variants with an allele frequency (VAF) of lower than 10% were excluded in the analysis of shared and distinct somatic mutations. We accepted variants as somatic events in the lymphoma cells even when they occurred in some reads (less than 3% VAF) of NTCs, as these are likely rare cell isolation contaminations and not shared germline mutations, for which the VAF should be close to 50%. In the analysis of constitutional variants, the VAF cutoff was set at 30% for both tumor cells and NTCs. Further analyses were performed in R, using the packages maftools (version 2.18.0) and dplyr (version 1.1.4), and Excel. Additionally, Control-FREEC (version 11.6) was used to process copy number variations and mosdepth (version 0.3.9) was used to calculate coverage data.

Owing to sequencing three independent libraries in the strategy using WGA, cases 2 and 3 provide an internal validation, as only mutations that are identified in at least 2/3 independently sequenced libraries are called. While in the other cases, no WGA was performed, the facts that we identified typical mutations in each case, and that we also sequenced NTCs in each case to eliminate germline variants and potential sequencing artifacts indicates that the results of our analyses are reliable and our method is viable for identifying shared and distinct somatic mutations in WES data of composite lymphomas.

**Interpretation of constitutional variants**

We considered variants as constitutional if they were detected in all three cell types of a patient with a VAF of at least 30% in tumour cells and NTCs. VCF files of theses constitutional variants of each of the six cases were imported into Emedgene (Ilumina, San Diego, CA, USA), an artificial intelligence (AI) based variant prioritization platform. Human Phenotype Ontology (HPO) terms “lymphoma”, "immunodeficiency" and "immune dysregulation" were used to identify variants with a high probability to the related diseases. Subsequently, prioritized variants were interpreted manually using multiple genomic databases like ClinVar (<https://www.ncbi.nlm.nih.gov/clinvar/>, accessed on 08 October 2024), gnomAD v4.1.0 (<https://gnomad.broadinstitute.org/>, accessed on 08 October 2024) and OMIM (<https://www.omim.org/>). Variant effects were also predicted by *in silico* prediction tools including AlphaMissense (<https://alphamissense.hegelab.org/search>), REVEL, MetaLR and MetaSVM. Variants were annotated using the dbNSFP42a database in ANNOVAR [5], which includes whole-exome SIFT, PolyPhen2, LRT, MutationTaster, PROVEAN, M-CAP, CADD and fathmm-MKL scores from dbNSFP version 4.2a. Splice site effects of variants were predicted by SpliceAI (<https://spliceailookup.broadinstitute.org/>).

REFERENCES

1. Lorenzi L, Lonardi S, Bonezzi M, Zini S, Bugatti M, Valzelli A, et al. Immunoglobulin light chain transcript detection by ultrasensitive RNA in situ hybridization for B-cell lymphoma diagnosis. *Virchows Archiv* 2024; **485**: 43–51.

2. Ventura RA, Martin-Subero JI, Jones M, McParland J, Gesk S, Mason DY, et al. FISH analysis for the detection of lymphoma-associated chromosomal abnormalities in routine paraffin-embedded tissue. *The Journal of Molecular Diagnostics JMD* 2006; **8**: 141–151.

3. Giefing M, Sawicz G, Siebert R. FISH and FICTION in Lymphoma Research. *Methods in Molecular Biology (Clifton, N.J.)* 2025; **2865**: 221–240.

4. Küppers R, Schneider M, Hansmann M-L. Laser-based microdissection of single cells from tissue sections and PCR analysis of rearranged immunoglobulin genes from isolated normal and malignant human B cells. *Methods in Molecular Biology (Clifton, N.J.)* 2019; **1956**: 61–75.

5. Wang K, Li M, Hakonarson H. ANNOVAR: functional annotation of genetic variants from high-throughput sequencing data. *Nucleic Acids Research* 2010; **38**: e164.


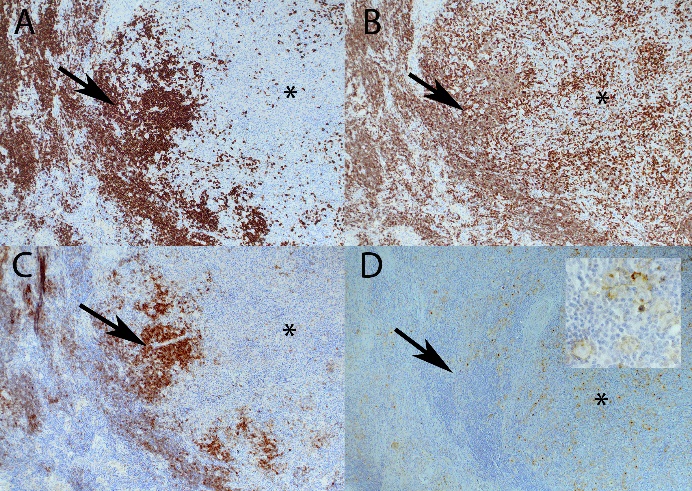

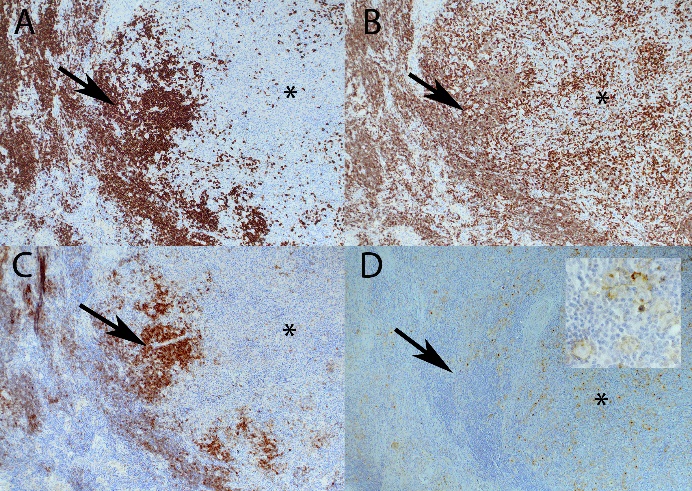

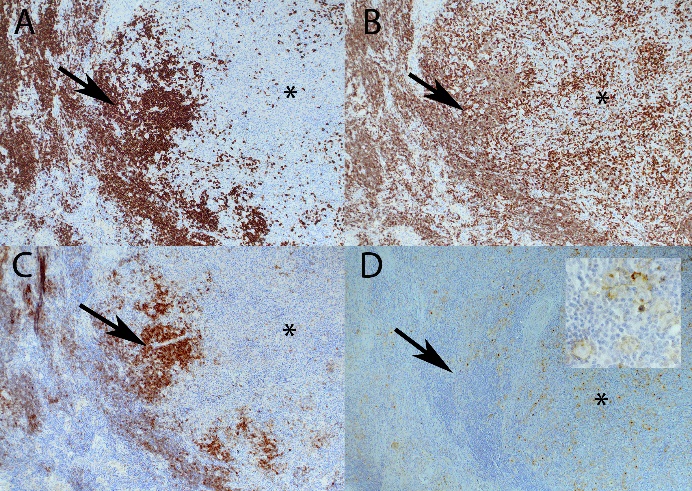

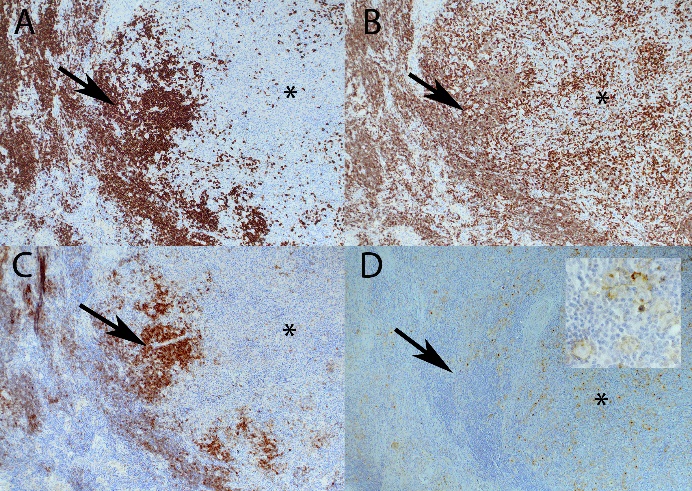


**Supplementary Figure S1. Histological evaluation of case 1, a composite CLL and classic HL.** Stainings for a) CD20, b) CD5, c) CD23, d) CD30. Insert shows HRS cells in a higher magnification. Arrows indicate small CLL cells with co-expression of CD20, CD5 and CD23. Asterisks mark the area containing HRS cells.

**
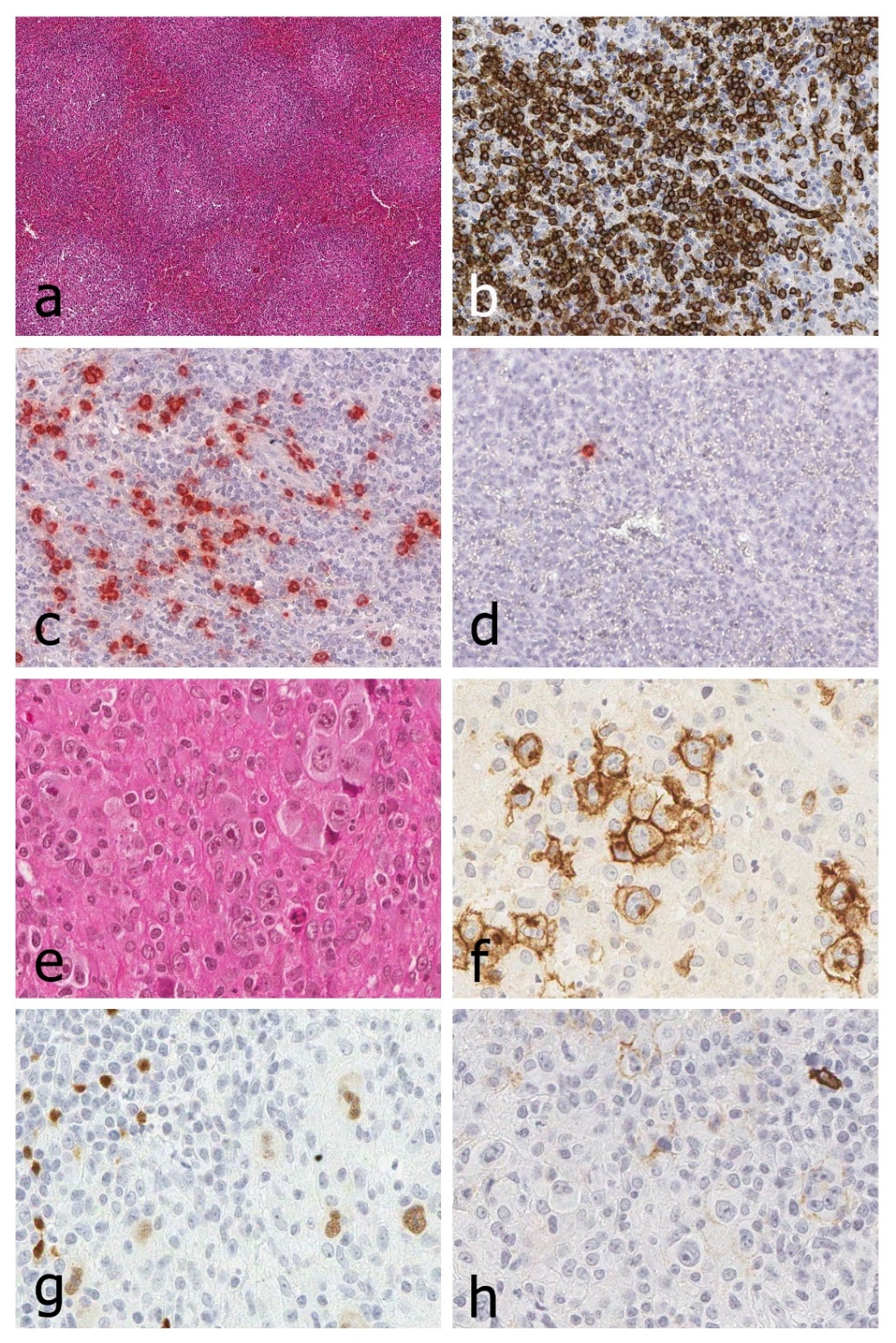
**

**Supplementary Figure S2. Histological evaluation of case 2, a composite SMZL (a-d) and classic HL (e-h).** a) A hematoxylin/eosin (HE) stain of the spleen showed effaced architecture with nodular infiltrate of small B lymphocytes that involved both white and red pulp, in the latter with evident intrasinusoidal distribution (CD20, b). The infiltrate was monotypic for kappa light chain transcript, as shown by RNAscope (c: kappa; d: lambda). e) HE staining revealed atypical, large, HRS cells in the second sample. HRS cells displayed strong CD30 (f), weak PAX5 (g) and weak to negative CD20 expression (h).

**
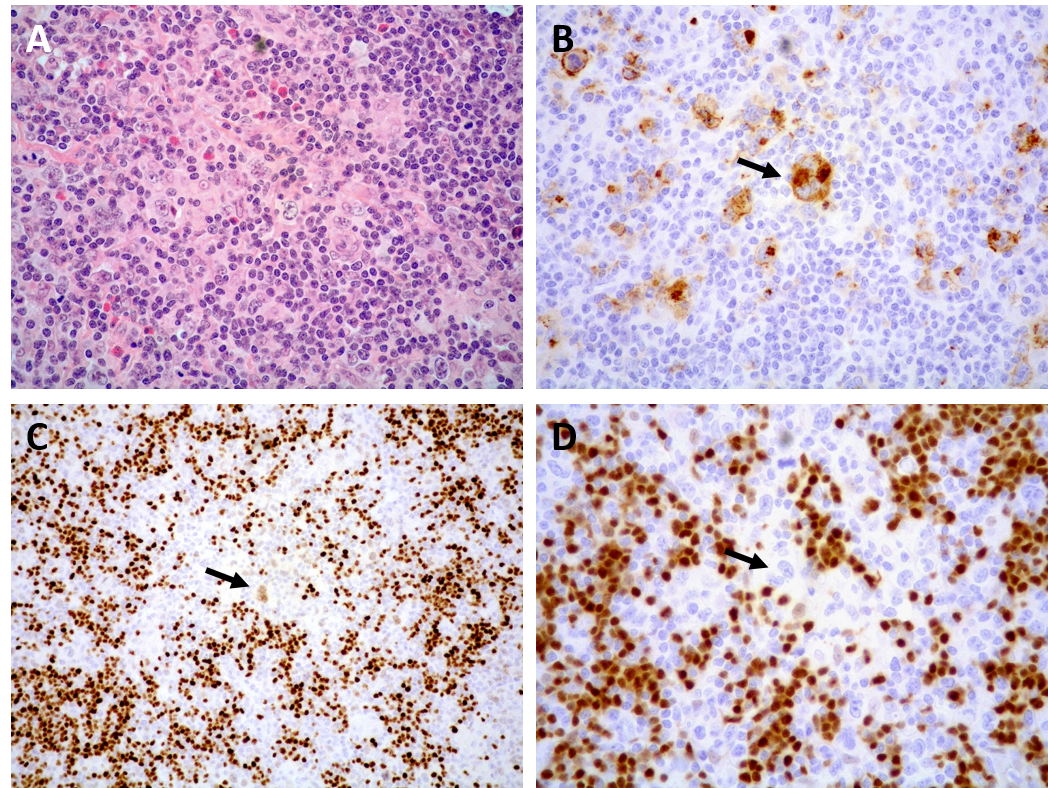
**

**Supplementary Figure S3. Histological evaluation of case 3, a composite MCL and classic HL.** a) HE staining, showing some HRS cells admixed with mature lymphocytes and a few eosinophils. b) CD15 staining, labeling a HRS cell in the middle (black arrow). c) PAX5 staining, showing that several mature lymphocytes are B cells, with a HRS cell in the middle that has the expected fainter staining (black arrow). d) Cyclin-D1 staining, showing that the mature B cells are MCL cells while a HRS cell in the middle is negative (black arrow).


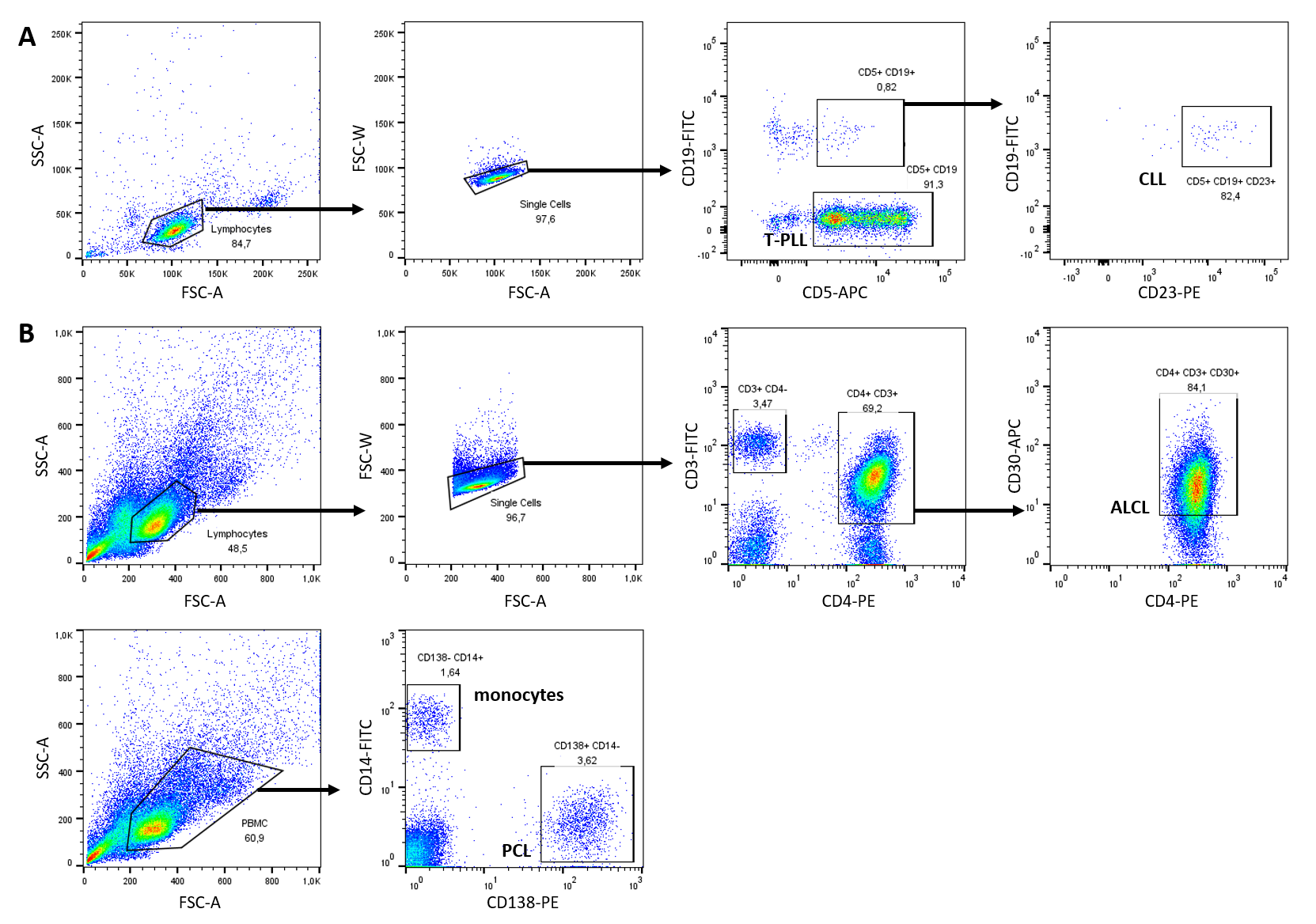


**Supplementary Figure S4. Flow-cytometric sorting strategies of combined B-NHL/T-NHL cases.**

Flow cytometry plots of PBMCs from cases 5 and 6. A) shows sort strategy of case 5, sorting T-PLL as CD19^-^CD5^+^ lymphocytes, and CLL as CD19^+^CD5^+^CD23^+^ lymphocytes. B) shows sort strategy of case 6, divided into two flow cytometry panels. ALCL cells are identified as CD4^+^CD3^+^CD30^+^ cells, whereas PCL cells are sorted as CD138^+^CD14^-^ cells, and monocytes, representing non-tumor cells, are sorted as CD138^-^CD14^+^ cells.

**
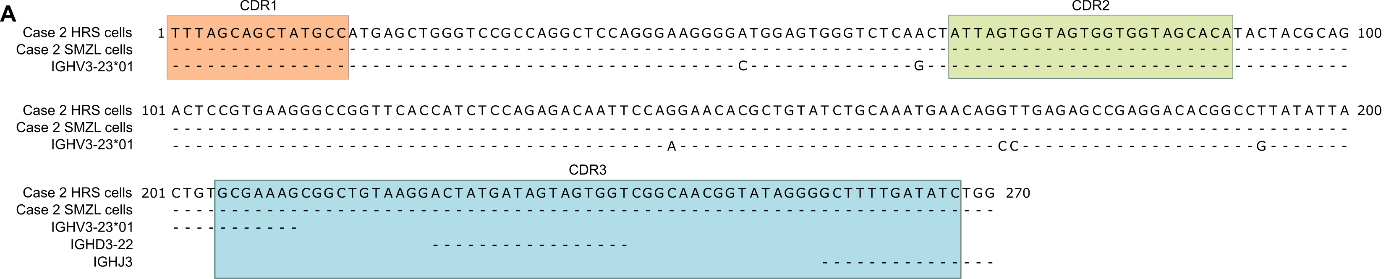

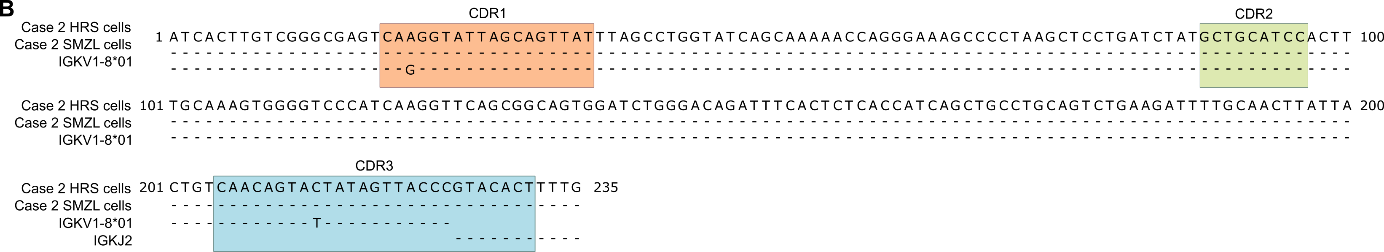

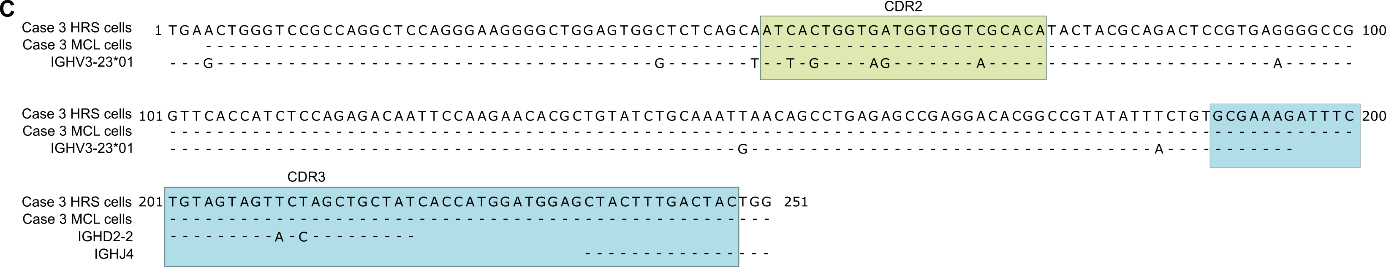

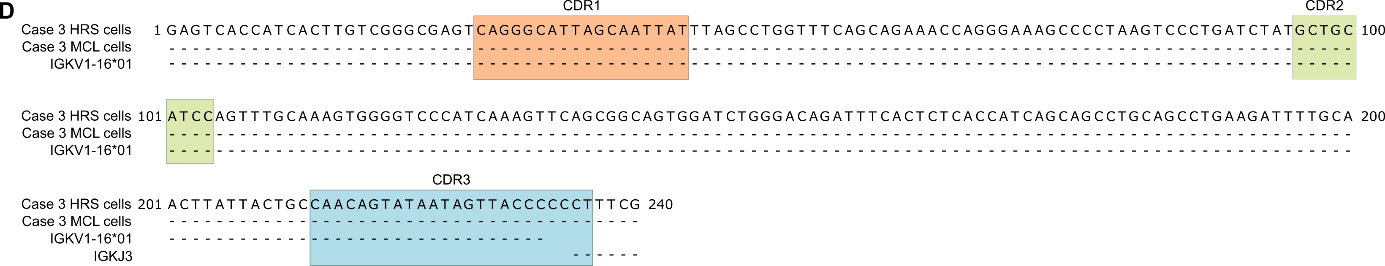

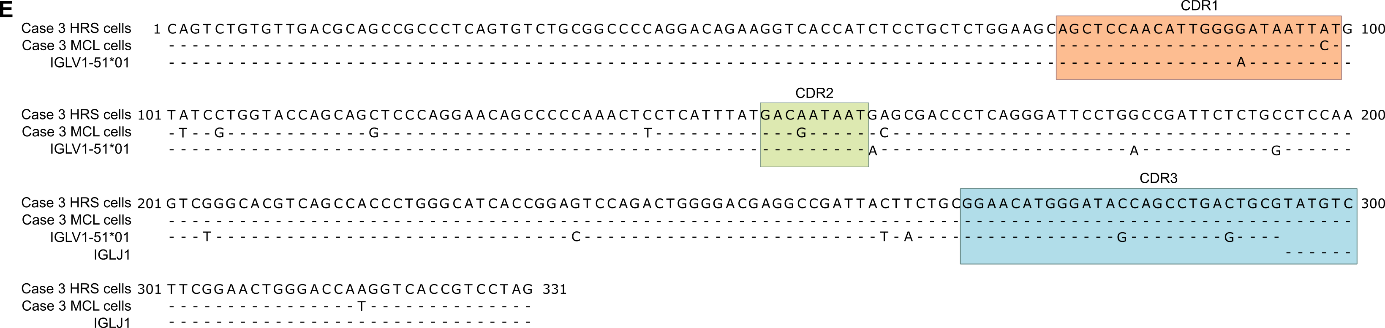

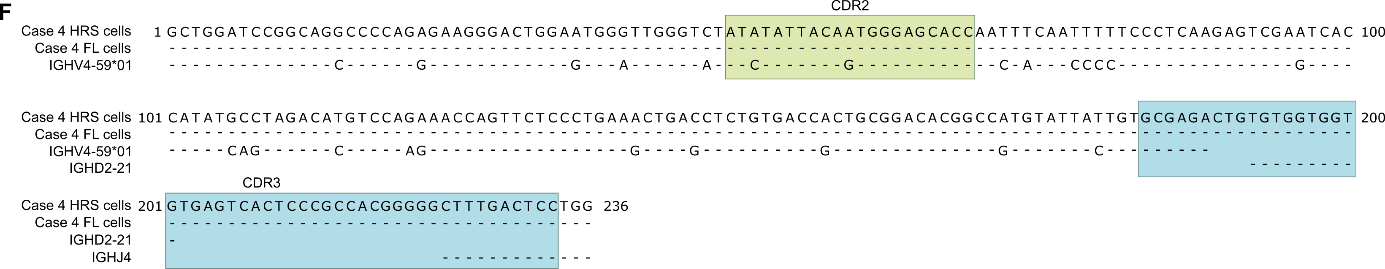

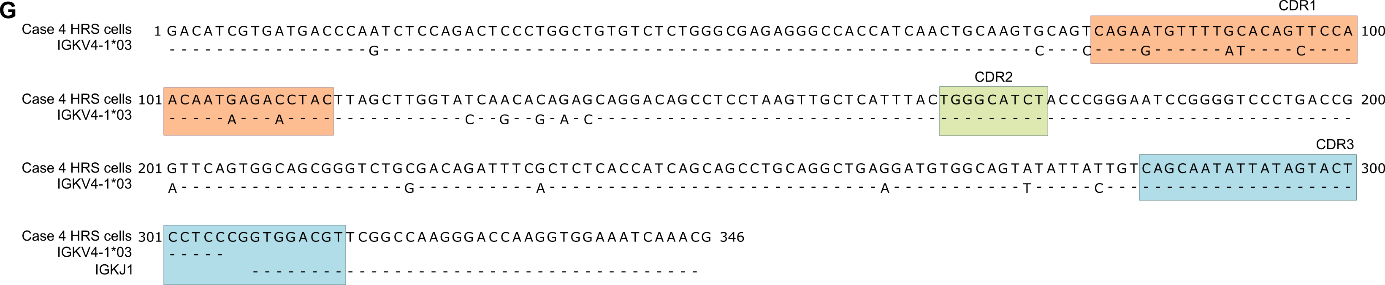
**

**Supplementary Figure S5.** **IGV gene sequences of combined lymphomas.** Shown are sequence comparisons of the rearranged IGHV, IGKV and IGLV rearrangements of the combined lymphomas to the most homologous germline IGV, IGHD, and IGJ genes. a) Case 2, IGHV3-23 rearrangement, b) Case 2, IGKV1-8 rearrangement, c) Case 3, IGHV3-23 rearrangement, d) Case 3, IGKV1-16 rearrangement, e) Case 3, IGLV1-51 rearrangement, f) Case 4, IGHV5-49 rearrangement, g) Case 4, IGKV4-1 rearrangement. The locations of the complementarity determining regions (CDR) 1-3 are indicated. Lines indicate sequence identity to the upper sequence.

**a)
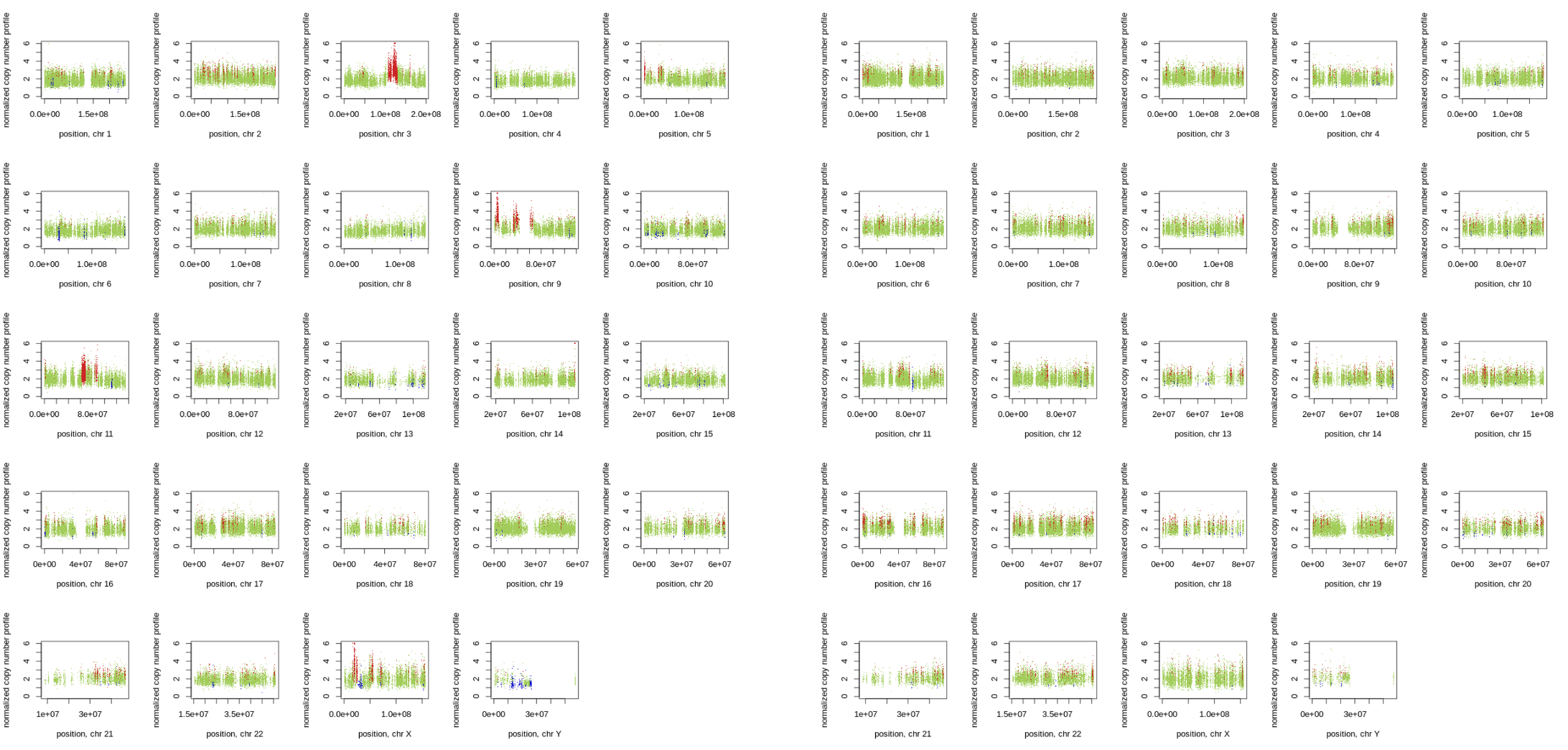
**

**case 1 CLL**

**case 1 HL**

**b)
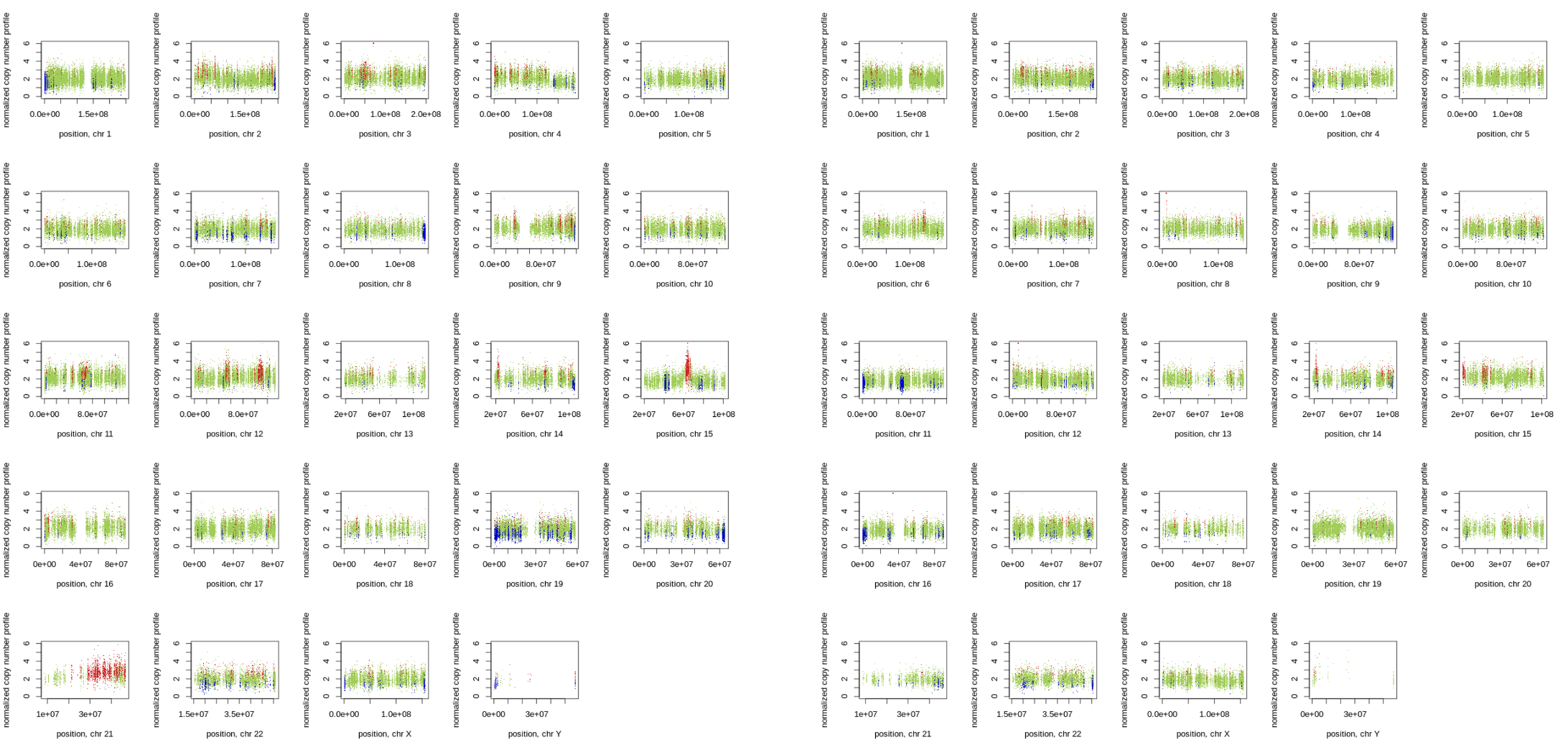
**

**case 4 HL**

**case 4 FL**

**c)
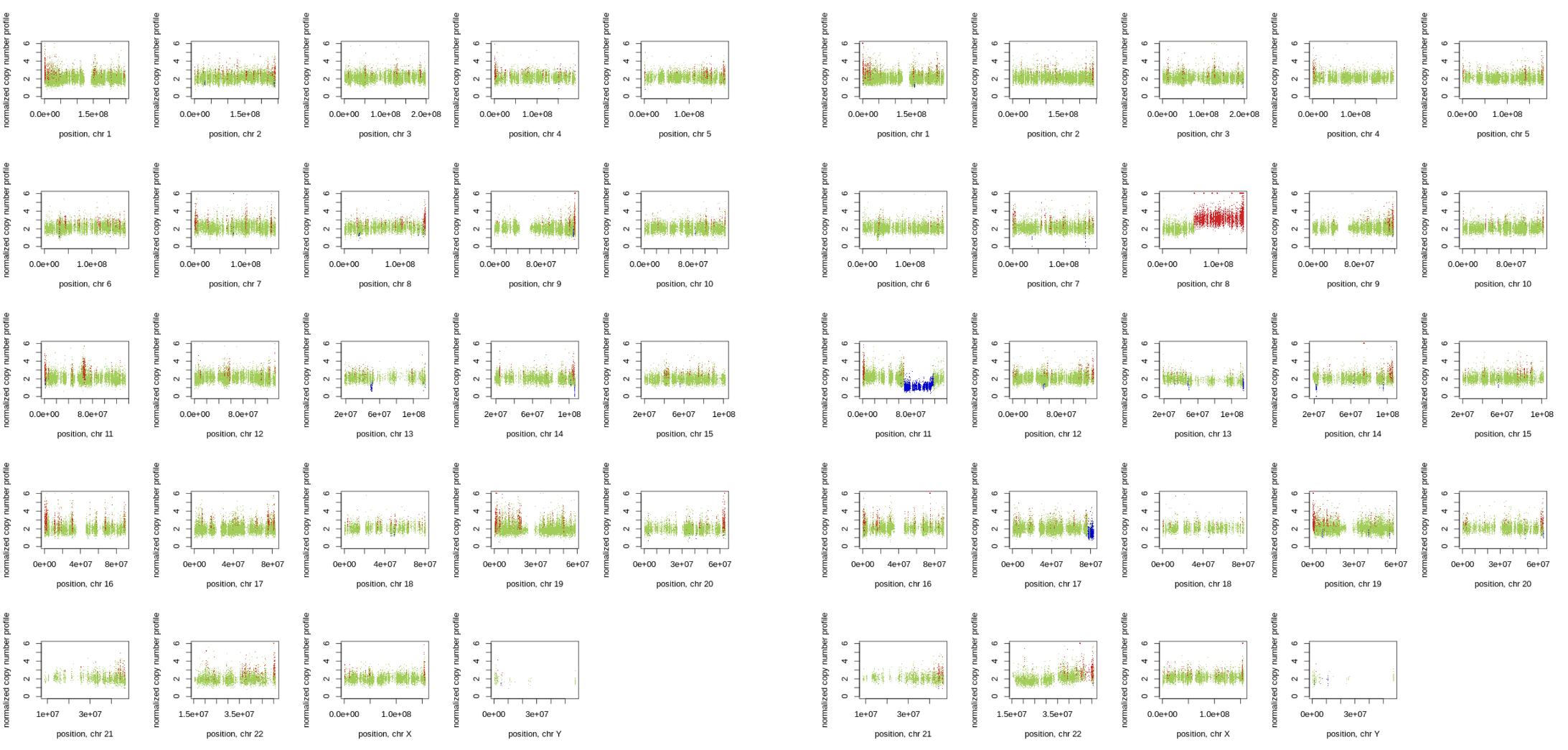
d)
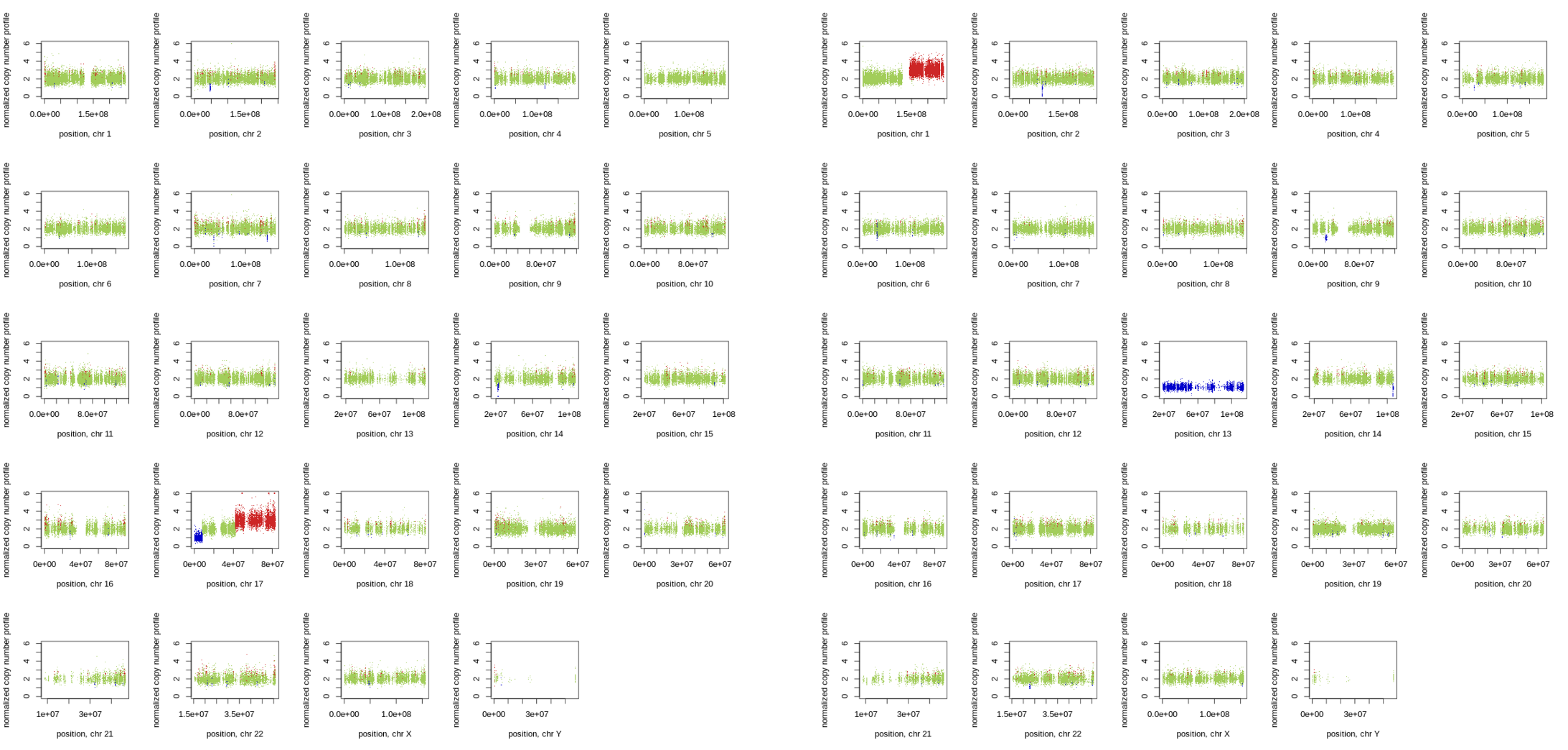
**

**case 5 T-PLL**

**case 5 CLL**

**case 6 PCL**

**case 6 ALCL**

**Supplementary Figure S6. Copy number variations in composite lymphoma cases.** Deletions are displayed in blue, amplifications are displayed in red. a) case 1, combined HL/CLL. b) case 4, combined HL/FL. c) case 5, combined CLL/T-PLL. d) case 6, combined PCL/ALCL.

**Suppl. Table S1: Antibodies used for Laser microdissection immunohistochemistry.**

| **Antibody** | **Clone** | **Species & Isotype** | **Cell/Tumor type** |
| --- | --- | --- | --- |
| anti-human CD3 |  | rabbit polyclonal | NTC |
| anti-human CD30 | BerH2 | mouse monoclonal | cHL |
| anti-human CD23 | DAK-CD23 | mouse monoclonal | CLL |
| anti-human BCL6 | PG-B6p | mouse monoclonal | FL |
| anti-human CCND1 | EP12 | rabbit monoclonal | MCL |

Antibodies were procured from Agilent (Santa Clara, CA, USA) under the Dako label.

**Suppl. Table S2:** **Antibodies used for flow cytometry.**

| **Antibody** | **Conjugate** | **Clone** | **Species & Isotype** | **Company** | **Case** |
| --- | --- | --- | --- | --- | --- |
| anti-human CD3 | FITC | UCHT1 | Mouse IgG1 κ | BD | 6 |
| anti-human CD30 | APC | BerH8 | Mouse IgG1 κ | BD | 6 |
| anti-human CD4 | PE | RPA-T4 | Mouse IgG1 κ | BD | 6 |
| anti-human CD14 | FITC | M5E2 | Mouse IgG2a κ | BD | 6 |
| anti-human CD138 | PE | MI15 | Mouse IgG1 κ | BD | 6 |
| anti-human CD5 | APC | UCHT2 | Mouse IgG1 κ | Biolegend | 5 |
| anti-human CD19 | FITC | HIB19 | Mouse IgG1 κ | BD | 5 |
| anti-human CD23 | PE | M-L233 | Mouse IgG1 κ | BD | 5 |

**Suppl. Table S3: Antibodies used for diagnostic immunohistochemistry.**

| **case** | | **antigen** | | **clone** | **host** | **dilution** | **manufacturer** |
| --- | --- | --- | --- | --- | --- | --- | --- |
| 1 | CD30 | | JCM182 | | Mouse IgG1 | ready to use | Leica |
| 1 | CD20 | | L26 | | Mouse IgG2a κ | ready to use | Leica |
| 1 | CD23 | | 1B12 | | Mouse IgG1 κ | ready to use | Leica |
| 1 | CD5 | | 4C7 | | Mouse IgG κ | ready to use | Leica |
| 2 | CD20 | | L26 | | Mouse IgG2a κ | 1:250 | Agilent |
| 2 | CD30 | | JCM182 | | Mouse IgG1 | 1:100 | Leica |
| 2 | PAX5 | | 24/Pax-5 | | Mouse IgG1 | 1:80 | BD |
| 3 | CD15 | | MMA | | Mouse IgM | ready to use | Leica |
| 3 | PAX5 | | 1E2W | | Mouse IgG1 | ready to use | Leica |
| 3 | CCND1 | | EP12 | | Rabbit IgG | ready to use | Leica |

**Suppl. Table S4. IGV gene analysis of composite B-cell lymphomas**

| **Case** | **Lymphoma/ leukemia** | **IGHV rearrangement** | **% mutation** | **Mutations identical** | **IGLV rearrangement** | **% mutation** | **Mutations identical** | **Clonally related** |
| --- | --- | --- | --- | --- | --- | --- | --- | --- |
| 1 | HL | IGHV1-18/IGHD2-21/IGHJ4 | 11.2 | n.a. | IGKV3-20/IGKJ2 | 5.9^†^ | n.a. | No |
|  | CLL | IGHV4-34/IGHD2-8/IGHJ3 | 0 |  | IGKV4-1/IGKJ2 | 0^†^ |  |  |
| 2 | HL | IGHV3-23/IGHD5-12/IGHJ3 | 2.8 | Yes | IGKV1-8/IGKJ2* | 0.9 | Yes | Yes |
|  | SMZL | IGHV3-23/IGHD5-12/IGHJ3 | 2.8 |  | IGKV1-8/IGKJ2* | 0.9 |  |  |
| 3 | HL | IGHV3-23/IGHD2-2/IGHJ4 | 5.6 | Yes | IGKV1-16/IGKJ3* | 0^#^ | Partially | Yes |
|  |  |  |  |  | IGLV1-51/IGLJ1** | 3.0^#^ |  |  |
|  | MCL | IGHV3-23/IGHD2-2/IGHJ4 | 5.6 |  | IGKV1-16/IGKJ3* | 0^#^ |  |  |
|  |  |  |  |  | IGLV1-51/IGLJ1** | 5.6^#^ |  |  |
| 4 | HL | IGHV4-59/IGHD2-21/IGHJ4 | 13.3 | Yes | IGKV4-1/IGKJ1** | 7.7 | n.a. | Yes |
|  | FL | IGHV4-59/IGHD2-21/IGHJ4 | 13.3 |  | - | - |  |  |
| 5 | CLL | IGHV3-30/IGHD6-19/IGHJ4**^§^ | 8 | n.a. |  | 2.6 | n.a. | n.a. |
|  |  | IGHV3-15/IGHD2-8/IGHJ4**^§^ | 8.2 |  | IGKV4-1/IGKJ2** |  |  |  |
|  | PCL | IGHV5-51/IGHD2-21/IGHJ4** | 2.4 | n.a. | Multiple rearrangements** | n.a. |  |  |
| 6 |  |  |  |  |  |  | n.a. | n.a. |

n.a., not applicable; ^†^unproductive; *verified by IgCaller in WES analysis; **identified by IgCaller in WES analysis;

^§^Two productive IG heavy chain gene rearrangements were identified. This could either mean that the CLL is biclonal, or that it is monoclonal, with productive IGHV genes on both heavy chain alleles. Examples for both of these configurations have been reported for CLL [61–63]. However, the WES analysis did not indicate a biclonal CLL, so it is likely that this is a monoclonal CLL with a biallelic IG heavy chain rearrangement.

^#^Finding a somatically mutated IGVL rearrangement and an unmutated IGKV region gene in a B cell is not unusual, because IGKV gene rearrangements are typically silenced by recombination of the kappa deleting element in Igl^+^ B cells, and thereby become exempted from being targeted by somatic hypermutation [64].

**Suppl. Table S5. Shared mutations**

| **case** | **Gene** | **position** | **type of mutation** | **protein effect** | **tumour VAF** | **NTC VAF** | **existing variant** | **SIFT** | **PolyPhen** |
| --- | --- | --- | --- | --- | --- | --- | --- | --- | --- |
| 2 | RBMXL2 | chr11:7089380 | missense | p.P87L | 0.143/0.368 (HL/SMZL) | 0.022 | rs750554717, COSV60980351 | deleterious (0.04) | possibly_damaging (0.634) |
| 2 | CA10 | chr17:51633638 | missense | p.R268C | 0.141/0.105 (HL/SMZL) | 0.006 | rs780641204, COSV53353383 | deleterious (0) | probably_damaging (1) |
| 2 | FARSA | chr19:12924251 | missense | p.V470M | 0.4/0.462 (HL/SMZL) | 0 | COSV53367759 | deleterious (0.04) | probably_damaging (0.94) |
| 2 | MAPRE3 | chr2:27025946 | missense | p.R231C | 0.121/0.125 (HL/SMZL) | 0 |  | deleterious (0) | probably_damaging (0.998) |
| 2 | MKRN1 | chr7:140456814 | missense | p.R275H | 0.308/0.176 (HL/SMZL) | 0 | rs376798349 | tolerated (0.08) | benign (0.066) |
| 3 | CDH8 | chr16:61653967 | missense | p.D681H | 0.243/0.48 (HL/MCL) | 0.005 |  | deleterious (0.01) | probably_damaging (1) |
| 3 | TP53 | chr17:7673802 | missense | p.R273H | 0.551/1 (HL/MCL) | 0.018 | rs28934576, CM920677, CM010472, CM004342, COSV52728930, COSV52676050, COSV52664805, COSV52660980 | tolerated (0.13) | possibly_damaging (0.643) |
| 3 | CLTC | chr17:59666206 | missense | p.R587L | 0.342/0.421 (HL/MCL) | 0 |  | deleterious (0) | possibly_damaging (0.613) |
| 3 | EML6 | chr2:54871596 | missense | p.D779N | 0.284/0.4 (HL/MCL) | 0.007 |  | tolerated (0.08) | possibly_damaging (0.786) |
| 3 | LMAN2L | chr2:96740032-96740034 | in-frame deletion | p.A3del | 0.418/0.476 (HL/MCL) | 0.008 |  |  |  |
| 3 | DOK5 | chr20:54591753 | missense | p.A183T | 0.301/0.588 (HL/MCL) | 0.015 | rs1485675129, COSV52821292 | deleterious (0.03) | benign (0.001) |
| 3 | DUSP22 | chr6:348938 | missense | p.T202M | 0.203/0.385 (HL/MCL) | 0.014 | rs571363943, COSV100739786 | deleterious (0.01) | benign (0) |
| 3 | GPR50 | chrX:151181049 | missense | p.K489M | 0.292/0.486 (HL/MCL) | 0.025 |  | deleterious_low confidence (0) | possibly_damaging (0.536) |
| 4 | ALDH1A3 | chr15:100900655 | missense | p.V322L | 0.421/0.167 (HL/FL) | 0.032 | CM163655 | deleterious (0.01) | probably_damaging (0.992) |
| 4 | MIDN | chr19:1255466 | missense | p.R301C | 0.267/0.211 (HL/FL) | 0 | rs1362021177, COSV56295627 | deleterious (0) | probably_damaging (0.994) |
| 4 | S1PR2 | chr19:10224596 | nonsense | p.Q104* | 0.25/0.194 (HL/FL) | 0 |  |  |  |
| 4 | CYP27C1 | chr2:127206078 | missense | p.R99W | 0.333/0.174 (HL/FL) | 0 |  | deleterious (0) | possibly_damaging (0.864) |
| 4 | CNTLN | chr9:17135288-17135289 | frameshift insertion | p.A75Dfs*48 | 0.278/0.115 (HL/FL) | 0 |  |  |  |
| 4 | CFP | chrX:47626142 | missense | p.T387N | 0.304/0.111 (HL/FL) | 0 |  | tolerated (0.33) | benign (0.335) |

**Suppl. Table S7. Selected constitutional variants**

| **affected gene** | **position** | **type of mutation** | **protein effect** | **VAF in NTCs** | **existing variants** | **ACMG-AMP criteria and classification** | **CADD** | **GnomAD allele frequency** | **AlphaMissense** |
| --- | --- | --- | --- | --- | --- | --- | --- | --- | --- |
| *CD27* | chr12:6445520 | missense | p.R78G | 0.469 | rs145433356 | PM2 (VUS) | 23.8 | 0.0001 | 0.166 (likely benign) |
| *CREBBP* | chr16:3781229 | missense | p.L551I | 0.644 | rs61753381, COSV52134580 | PP2, BA1, BP4 (benign) | 24.3 | 0.007154 | 0.087 (likely benign) |
| *CREBBP* | chr16:3729277 | missense | p.V1924M | 0.687 | rs368145743 | PP2, BS2, BP4 (likely benign) | 23.9 | 0.000445 | 0.119 (likely benign)^#^ |
| *FANCA* | chr16:89771678 | missense | p.M717I | 0.491 | rs1131660 | PP3, BA1 (benign), GC-HBOC classification: benign | 23.7 | 0.022344 | 0.176 (likely benign) |
| *GATA2* | chr3:128486117 | missense | p.P161A | 0.538 | rs34799090, COSV62007567 | BS1, BS2 (benign) | 19.35 | 0.008537 | 0.07 (likely benign) |
| *MYC* | chr8:127740678 | missense | p.S362F | 0.464 | rs200431478, COSV99420567 | BS2 (likely benign) | 27.8 | 0.000335 | 0.993 (likely pathogenic) |
| *SETD2* | chr3:47120671 | missense | p.R1322Q | 0.468 | rs147170912, COSV99042141 | PM2 (VUS) | 22.7 | 0.000007 | 0.103 (likely benign) |
| *TET2* | chr4:105275662 | missense | p.V1718L | 0.545 | rs142312318, COSV54398060 | BS2, BP4 (likely benign) | 0.254 | 0.004477 | 0.098 (likely benign) |
| *TRAF3IP2* | chr6:111566485 | missense | p.E479K | 0.391 | rs149504543 | PM2, BP4 (VUS) | 21.3 | 0.000093 | 0.995 (likely pathogenic) |
| *JAK3* | chr19:17834887 | missense | p.V722I | 0.475 | rs3213409, CM000172, COSV71685519 | BS1, BS2, BP4 (benign) | 14.82 | 0.007432 | 0.071 (likely benign) |
| *DUSP22* | chr6:304648 | missense | p.I14M | 0.419 | COSV60528266 | PM2 (VUS) | 21 |  | 0.564 (likely pathogenic) |
| *NOD2* | chr16:50729867-50729868 | frameshift insertion |  | 0.333 |  | PVS1_Sup, PS3_ Sup, BS2 (VUS) |  |  |  |
| *ARID1A* | chr1:26763012 | missense | p.N820S | 0.555 | rs773242876, COSV99055395 | PM2 (VUS) | 23.8 | 0.000007 | 0.086 (likely benign) |
| *BRCA2* | chr13:32398184-32398185 | frameshift insertion | p.I3224fs | 0.333 | CI022172 | PVS1, PS4, PM2_SUP (pathogenic), GC-HBOC classification: pathogenic | 28.5 |  |  |
| *ATM* | chr11:108335880 | missense | p.Q2729H | 0.382 | rs587781946, CM143104, COSV53762213, COSV99586681 | PM2, PP3 (VUS)/GC-HBOC classification: (VUS) | 23.7 | 0.000016 | 0.976 (likely pathogenic) |
| *BTK* | chrX:101390479 | missense | p.E24G | 0.706 | rs5951308, COSV58121598 | BA1, BP4 (benign) | 4.83 | 0.338641 | 0.996 (likely pathogenic) |
| *NOTCH2* | chr1:119925578 | missense | p.L1413H | 0.4 | rs41313282, COSV56681418 | PP2, BS1, BS2, BP4 (benign) | 19.01 | 0.003746 | 0.07 (likely benign) |
| *SETD2* | chr3:47122481 | missense | p.N719D | 0.357 | rs115859828, COSV57453200 | PM2 (VUS) | 23 | 0.00005 | 0.134 (likely benign) |
| *ATM* | chr11:108254034 | missense | p.S707P | 1 | rs4986761, CM013692, COSV53743430 | BS1, BS2, BP4 (benign), GC-HBOC classification: benign | 11.04 | 0.007818 | 0.08 (likely benign) |
| *BRCA1* | chr17:43092412 | missense | p.S1040N | 0.371 | rs4986852, CM940175, COSV58791858 | BA1,BS3_Mod, BP4 (benign), GC-HBOC classification: benign | 14.02 | 0.013154* | 0.3 (likely benign) |
| *BRCA1* | chr17:43094045 | missense | p.R496C | 0.530 | rs28897676, CM065005, COSV58797441 | BS1,BS2, BP4 (benign), GC-HBOC classification: benign | 4.7 | 0.000235* | 0.08 (likely benign) |
| *IBTK* | chr6:82191202 | missense | p.H1134R | 0.426 | rs145495116 | BS1, BP4 (likely benign) | 15.48 | 0.000513 | 0.076 (likely benign) |
| *MCL1* | chr1:150578851 | missense | p.A227V | 0.555 | rs11580946, COSV57191585 | BS1, BS2 (benign) | 25.9 | 0.008742 | 0.919 (likely pathogenic) |
| *NFKB1* | chr4:102597543 | missense | p.M507V | 0.5 | rs4648072 | PP2, BS1, BS2, BP4 (benign) | 8.69 | 0.018841 | 0.227 (likely benign) |
| *PTPN13* | chr4:86809827 | missense | p.T2386I | 0.392 | rs61730641, COSV57419424 | BS1, BS2 (benign) | 24.1 | 0.012955** | 0.373 (ambiguous) |
| *PTPN13* | chr4:86803817 | missense | p.R2210Q | 0.449 | rs61750816, COSV57401812 | BA1 (benign) | 18.28 | 0.021763 |  |
| *EP300* | chr22:41150154 | missense | p.P925S | 0.667 | rs148884710, COSV54339374, COSV54337074 | PM2, BP4 (VUS) | 20.3 | 0.004099 | 0.099 (likely benign) |
| *KMT2C*/*MLL3* | chr7:152163145 | missense | p.Q3478E | 0.413 | rs142835638, COSV51503272 | BS1, BS2 (benign) | 22.8 | 0.00303 | 0.121 (likely benign) |
| *MTOR* | chr1:11247950 | missense | p.A329T | 0.568 | rs35903812, COSV63870597 | BS1, BS2 (benign) | 23.4 | 0.002625 | 0.086 (likely benign) |
| *NOD2* | chr16:50722629 | missense | p.G908R | 0.627 | rs2066845, CM011829 | Risk of Crohn's disease, PS4_Sup, BS1, BS2 (benign) | 26.3 | 0.01424** | 0.85 (likely pathogenic) |
| *S1PR2* | chr19:10224876 | missense | p.N10L | 0.5 | rs56357614 | BS1, BS2, BP4 (benign) | 16.37 | 0.008214 | 0.385 (ambiguous) |

*GnomAD_Exome

**ALFA
